# Prevalence and risk factors of overactive bladder syndrome among Egyptian medical students, and its impact on health-related quality of life, cross-sectional study

**DOI:** 10.1038/s41598-026-53181-4

**Published:** 2026-05-27

**Authors:** Ahmed Fayez Mohamed, Abdelmonam M. Hagag, May Mahmoud Elgamal, Ibrahim Essam Elshaikh, Asmaa Hamdy Abdellatif, Abdullah Ali, Ahmed M. Eliwa

**Affiliations:** 1 Faculty of Medicine, Merit University, Tell Thamud, New Qassasin, Ismailia, New Sohag, Egypt; 2 Faculty of Medicine, Zagazig University, Zagazig, Egypt; 3 Faculty of Medicine, Kafr Elsheikh University, Kafr Elsheikh, Egypt; 4 Faculty of Medicine, Qena University, Qena, Egypt; 5 Faculty of Medicine, Mansoura University, Mansoura, Egypt; 6 Zagazig University, Zagazig, Egypt; 7 Medical Research Group of Egypt, Negida Academy Llc, 148 Massachusetts Avenue, Avenuearlington, MA 02474 USA

**Keywords:** Overactive bladder, OAB, Health-related quality of life, Cross-sectional study, Diseases, Health care, Medical research, Risk factors, Urology

## Abstract

Overactive bladder (OAB) is a common chronic condition that can have a significant impact on quality of life (HRQL). We aimed to assess the prevalence of OAB bother and its related quality of life among medical students in Egypt. A cross-sectional study among medical students in Egypt. An online questionnaire was shared via online platforms to collect the intended participants. We used the Overactive Bladder Symptoms and Health-Related Quality of Life Short Form (OAB-q SF). We assessed the difference between participants in their baseline characteristics using the Mann–Whitney U and Kruskal–Wallis Tests. We performed a multiple linear regression to assess the potential risk factors. 1003 participants completed the questionnaire. The median transformed OAB bother score was 10[3.33, 23.33], while the median transformed score of HRQL was 93.8[80, 98.46]. The prevalence was about 15%, while about 51.65% reported a decline in HRQL. A statistically significant association between OAB bother and academic phase, energy drink, or satisfaction with social life was reported. We found no association between OAB bother and caffeinated drinks or smoking. Finally, neither age, gender, nor BMI had an association with OAB bother. A low prevalence of OAB among medical students was found. Consumption of Energy drinks was found to be a risk factor, while caffeinated drinks had no effect. OAB was found to be higher in the academic phase than in the clinical one. Several studies should be conducted to further assess the risk factors of overactive bladder.

Clinicaltrials.gov registration: NCT07044778, June 2025.

**Supplementary Information:**

The online version contains supplementary material available at 10.1038/s41598-026-53181-4.

## Introduction

Overactive bladder (OAB) is a common chronic condition that can have a significant impact on quality of life. It is characterized by urinary urgency with or without urgency incontinence, and usually with an increase in daytime frequency alongside nocturia, coupled with or without urge incontinence, not resulting from infection or other detectable pathological conditions^1–5^. The cause of OAB is often idiopathic and multifactorial. Possible mechanisms include urothelial dysfunction, detrusor overactivity, autonomic dysregulation, and altered afferent signaling, with some patients having comorbid associations of conditions such as metabolic syndrome, depression, and sleep disorders^6^.

It is diagnosed based on history and present symptoms after ruling out urinary tract infection and other causes, according to the American Urological Association (AUA) and the Society of Urodynamics, Female Pelvic Medicine & Urogenital Reconstruction (SUFU). The use of symptom questionnaires and voiding diaries is also recommended by the AUA/SUFU. Routine imaging is not recommended by the AUA/SUFU, except in cases of atypical presentation or treatment failure^2^.

The economic burden of OAB is significant. In the US, annual direct costs for one patient range from $656 to $860, with total costs exceeding $11,000 per year. It increases healthcare utilization and costs, especially in patients with comorbidities, and national costs have risen from $65.9 billion in 2007 to $82.6 billion in 2020^7,8^. Similarly, another study conducted in 5 European countries and Canada concluded that the total annual burden of overactive bladder was €9.7 billion^9^. In the Middle East, the economic burden is not clearly presented in systematic cost studies, which may be due to the lack of healthcare systems in these countries; however, the high global incidence and regional low treatment rates in these countries indicate a considerable and increasing economic burden in terms of direct and indirect costs, especially in low- middle income the Middle east countries, such as Egypt.

The prevalence of OAB varies significantly by population, gender, age, and study methodology. In the US, 10–20% of adults are affected, with higher rates in the elderly. A recent US study shows a prevalence of 14.5% in men and similar or higher rates in women, with higher rates in older adults^10–12^. Another study among the Polish population found an overall prevalence of OAB being 33.9%, with a significant difference between males (26.8%) and females (39.5%)^13^. A recent study found the overall prevalence of OAB to be 27.4% in a Middle Eastern population, with a higher rate in women than men^14^. Among medical students, OAB is significantly common, with a prevalence of 10–45% in recent studies^15,16^. Whereas OAB has been investigated among certain medical students, no study so far has been performed to assess this condition in Egyptian medical students.

OAB is not well-reported in Egypt, and thus, there is a lack of knowledge in the literature. Because previous studies among medical students in the Middle East showed a high prevalence rate, this study aimed to determine the prevalence of OAB among Egyptian medical students, identify the risk factors, and assess its impact on quality of life.

## Methodology

### Study design

This cross-sectional study was conducted among medical students in Egypt using an online self-reported questionnaire. We followed the STROBE guidelines in reporting the study^17^. Data was collected between May and June 2025. The study protocol was approved by the institutional review board (IRB), Zagazig University, Faculty of Medicine (approval number: ZU-IRB # 1328 /18-May-2025). The study was further registered in ClinicalTrials.gov under the number: NCT07044778. The study participants were included after providing informed consent.

### Eligibility criteria

We included medical students in Egypt, whose ages ranged from 18 to 27, who had access to the online questionnaire and agreed to participate in the study.

On the other hand, we excluded non-medical students, pharmacy, nursing, and dental students. We also excluded medical interns, or who refused to participate in the study.

### Sample size and sampling

We calculated the sample size based on the total medical students in Egypt, which is 121,320, according to the Central Agency for Public Mobilization and Statistics (CAPMAS) for the academic year 2023–2024, assuming a 44.5% expected prevalence of overactive bladder based on a previous study conducted on a similar population in Jordon^15^, a 95% confidence interval, 80% power, and 5% error margin, the required sample size was calculated using Epi Info version 7.2.60 to be 378, after adding a 10% non-response rate. The final sample size needed for this study became 416 students. A convenience sampling technique was used to recruit participants.

### Pilot testing of the questionnaire

The questionnaire was piloted among 40 medical students who did not participate in the main study. They were asked to assess the questionnaire in terms of readability, clarity, and overall understanding to ensure that all items were interpreted as intended.

### Data collection

Data were collected using an online questionnaire distributed via social media platforms, including WhatsApp, Twitter, Facebook, and Telegram. The form was distributed in these social media platforms via the study collaborators, and the target population was asked to voluntarily participate in the study by filling out the questionnaire. The questionnaire was divided into three parts; The first section collected sociodemographic data, including age, sex, and academic year. It also addressed life risk factors, such as body weight, presence of chronic disease, previous surgery, stressful life, and consumption of soft drinks^15^. The second and third sections included questions regarding the presence of overactive bladder symptoms and their related quality of life using the Overactive Bladder Symptoms and Health-Related Quality of Life Short Form (OAB-q SF), with knowledge that the OAB-q SF assesses symptom bother and health-related quality of life^18^. The Scale is divided into two sections. The first was a 6-item asks about the presence of overactive bladder symptoms in the past 4 weeks. Each question has 6 available answers, ranging from ‘not at all’, which indicates the absence of symptoms, to ‘a very great deal’, which indicates the presence of these symptoms. The score of each question ranges from 1 to 6, and the total score is 36. A score of 18 or more indicates the presence of overactive bladder symptoms^16^. The third section assessed the impact of overactive bladder on quality of life. This is a 13-item questionnaire, each one has 6 answers (None of the time, little of the time, some of the time, A good bit of the time, Most of the time, All the time). Each item is scored from 1 to 6, resulting in a total score range from 13 to78. We transformed the score for both OAB bother and HRQL according to Coyne et al. 2015^18^ as follows: For OAB bother: $\:\frac{(Actual\:raw\:score\:-lowest\:possible\:raw\:score\left(6\right))}{Possible\:raw\:score\:range\:\left(30\right)}*100$. For the HQRL: $\:\frac{(heighest\:possibel\:score\:\left(78\right)-acutal\:raw\:score)}{possible\:raw\:score\:range\left(65\right)}*100$

### Statistical analysis

Data analysis was performed using SPSS software version 29. Categorical variables were summarized using frequencies and percentages, while continuous variables were tested for normality. If data were normally distributed, the mean and standard deviation were used; otherwise, the median and interquartile range were reported.

To compare differences in overactive bladder (OAB) symptoms and health-related quality of life (HRQL) scores across different demographic and behavioral characteristics (such as gender, academic phase, smoking status, and beverage consumption), non-parametric tests were applied. Specifically, the Mann–Whitney U and Kruskal–Wallis Tests were used to compare the median OAB bother and HRQL scores between two independent groups.

Correlation between the severity of OAB symptoms and quality of life was assessed using Spearman’s rank correlation coefficient. Additionally, multiple linear regression analysis was conducted to examine the association between overactive bladder symptoms (OAB) and health-related quality of life (HRQL) scores, as well as to explore potential risk factors associated with the presence of OAB symptoms. These included variables such as history of urinary tract infections (UTIS), consumption of soft drinks, coffee, or tea. Finally, a p-value of less than 0.05 was considered statistically significant throughout the analysis.

## Results

### Sociodemographic data

**Fig. 1:**
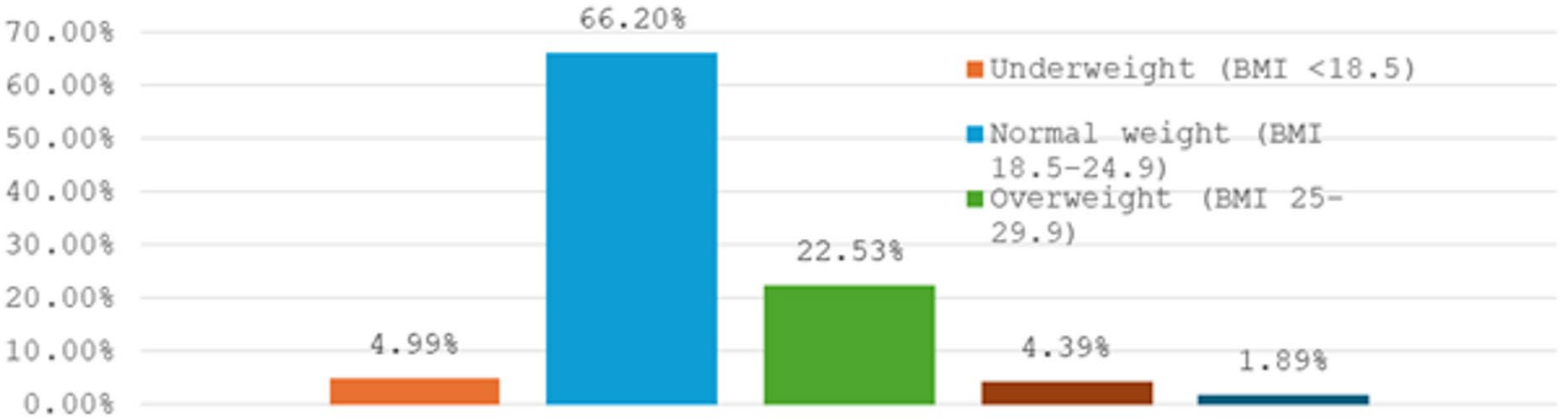
Distribution of BMI among medical students.

From May 18, 2025, to June 15, 2025, 1138 individuals filled out the questionnaire, of which 1003 were eligible, and the remaining 135 were excluded. The students’ mean age was 21.57 ± 1.684. 51.5% (513 students) of the students were males, and 74.18% (744) were from the clinical phases (academic years from 3 to 5). 664 students (66.4%) were of normal weight, 226 (22.53%) were overweight, while 44, 19, and 50 students were obese, morbidly obese, and underweight, respectively. See Fig. 1.

544 students were satisfied with their academic performance, while only 153 were satisfied with their social life. Additionally, 166 (16.55%) students usually had energy drinks, 550 (54.84%) students usually had coffee, and 677 (67.50%) students usually had tea. See Table 1.

**Table 1:** Sociodemographic data between medical students.

		*N*	%
Age	Mean ± SD	21.57 ± 1.684	
Gender	Female	490	48.85%
	Male	513	51.15%
What is your current academic phase	Academic phase	259	25.82%
	Clinical phase	744	74.18%
What is your current academic year	First year	142	14.16%
	Second year	117	11.67%
	Third year	229	22.83%
	Fourth year	148	14.76%
	Fifth year	367	36.59%
What is your body weight	Underweight (BMI < 18.5)	50	4.99%
	Normal weight (BMI 18.5–24.9)	664	66.20%
	Overweight (BMI 25–29.9)	226	22.53%
	Obese (BMI 30–34.9)	44	4.39%
	Morbidly obese (BMI ≥ 35)	19	1.89%
Do you have a chronic disease?	No	933	93.02%
	Yes	70	6.98%
Have you had previous surgery?	No	827	82.45%
	Yes	176	17.55%
Do you have a history of trauma?	No	910	90.73%
	Yes	93	9.27%
Rate your satisfaction with your academic performance	Very satisfied	94	9.37%
	Satisfied	450	44.87%
	Some-what	319	31.80%
	Unsatisfied	111	11.07%
	Very unsatisfied	29	2.89%
Rate your satisfaction with your social life	Very satisfied	124	12.36%
	Satisfied	434	43.27%
	Some-what	314	31.31%
	Unsatisfied	109	10.87%
	Very unsatisfied	22	2.19%
Rate your stress level	1	84	8.37%
	2	115	11.47%
	3	364	36.29%
	4	286	28.51%
	5	154	15.35%
Do you have a chronic disease?	No	933	93%
	Yes	70	7%
Do you have a previous surgery?	No	827	82.5%
	Yes	176	17.5%
Do you have a history of trauma?	No	910	90.7%
	Yes	93	9.3%
Are you a smoker?	No	954	95.11%
	Yes	49	4.89%
Do you usually have energy drinks?	No	837	83.45%
	Yes	166	16.55%
Do you usually have coffee?	No	453	45.16%
	Yes	550	54.84%
Do you usually have tea?	No	326	32.50%
	Yes	677	67.50%
Overactive bladder symptoms transformed score	Median [IQR]	10 [3.33, 23.33]	
HRQL transformed score	Median [IQR]	93.8 [80, 98.46]	
Overactive bladder symptoms bother	No	850	84.70%
	Yes	153	15.30%
HRQL decrease	No	485	48.35%
	Yes	518	51.65%

### Overactive bladder symptoms bother (OAB bother) and its health-related quality of life (HRQL)

**Fig. 2:**
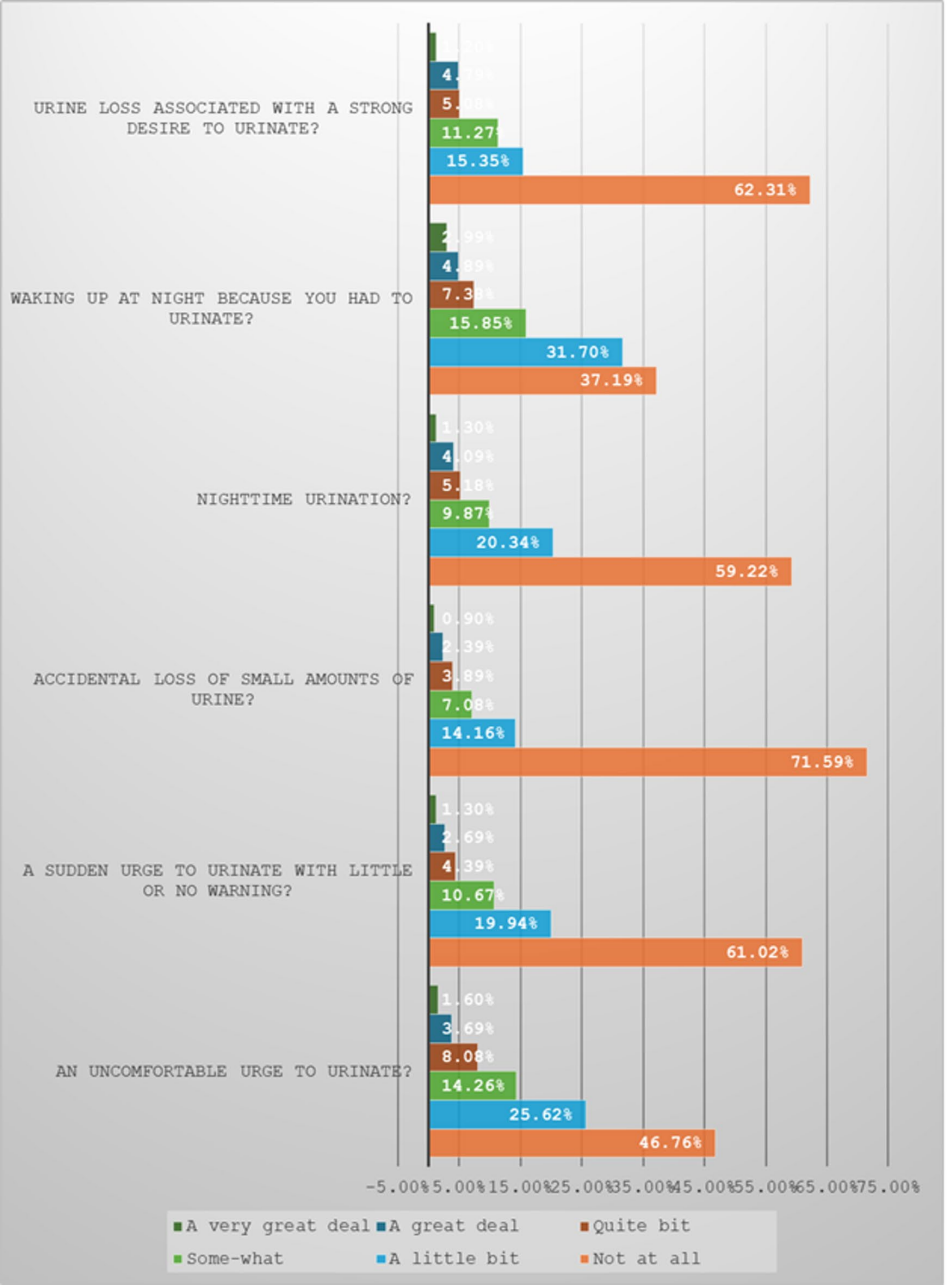
Overactive bladder symptoms bother.

The median transformed score for the overactive bladder symptoms bother was 10 [3.33, 23.33]. As shown in Fig. 2, most students did not have most of the overactive bladder symptoms bother. See Fig. 2. About 18.6% of students had an uncomfortable urge to urinate; its severity ranges from some-what to a great deal. 16.4% had a sudden urge to urinate, and 14.3% had an accidental loss of a small amount of urine. Additionally, about 20.5% had a nocturia, 31.2% usually woke up at night due to urination, and 21.4% usually lost urine when they had a strong desire to urinate.

On the other hand, the median transformed score for the health-related quality of life (HRQL) was 93.8 [80, 98.46]. Again, most students were not affected by overactive bladder symptoms. See Table 2.

**Table 2:** Health-related quality of life (HRQL).

Symptoms	None of the time	A little of the time	Some of the time	A good bit of the time	Most of the time	All of the time
Caused you to plan ‘‘escape routes’’ to restrooms in public places?	626 (62.4%)	202 (20.1%)	108 (10.8%)	35 (3.5%)	25 (2.5%)	7 (0.7%)
Made you feel like there is something wrong with you?	553 (55.1%)	229 (22.8%)	125 (12.5%)	45 (4.5%)	30 (3%)	21 (2.1%)
Interfered with your ability to get a good night’s rest?	567 (56.5%)	222 (22.1%)	121 (12.1%)	43 (4.3%)	36 (3.6%)	14 (1.4%)
Made you frustrated or annoyed about the amount of time you spend in the restroom?	587 (58.5%)	216 (21.5%)	115 (11.5%)	48 (4.8%)	26 (2.6%)	11 (1.1%)
Made you avoid activities away from restrooms (i.e., walks, running, hiking)?	721 (71.9%)	135 (13.5%)	81 (8.1%)	37 (3.7%)	18 (1.8%)	11 (1.1%)
Awakened you during sleep?	469 (46.8%)	301 (30%)	125 (12.5%)	57 (5.7%)	39 (3.9%)	12 (1.2%)
Caused you to decrease your physical activities (exercising, sports, etc.)?	743 (74.%)	121 (12.1%)	81 (8.1%)	28 (2.8%)	20 (2%)	10 (1%)
Caused you to have problems with your partner or spouse?	830 (82.8%)	77 (7.7%)	54 (5.4%)	23 (2.3%)	11 (1.1%)	8 (0.8%)
Made you uncomfortable while traveling with others because of needing to stop for a restroom?	636 (63.4%)	183 (18.2%)	105 (10.5%)	41 (4.1%)	23 (2.3%)	15 (1.5%)
Affected your relationships with family and friends?	793 (79.1%)	94 (9.4%)	60 (6%)	26 (2.6%)	22 (2.2%)	8 (0.8%)
Interfered with getting the amount of sleep you needed?	656 (65.4%)	172 (17.1%)	88 (8.8%)	49 (4.9%)	21 (2.1%)	17 (1.7%)
Caused you embarrassment?	685 (68.3%)	157 (15.7%)	97 (9.7%)	37 (3.7%)	15 (1.5%)	12 (1.2%)
Caused you to locate the closest restroom as soon as you arrive at a place you have never been?	669 (66.7%)	164 (16.4%)	102 (12.2%)	33 (3.3%)	22 (2.2%)	13 (1.3%)

### Binary comparison

For the overactive bladder symptoms bother transformed score, there was a statistically significant difference between studying phases, as the academic phase had higher score than clinical phase (median [IQR]: 13.33 [6.67, 30], and 10 [3.33, 23.33], P value: 0.003) for academic and clinical phases respectively, While there was no difference between gender and smoking, Energy drinking, coffee, or tea drinking status. See Table 3. When assessing the differences between these variables on each symptom of overactive bladder, we found statistically significant differences between gender in the sudden urge to urinate (P value:0.008). For the academic phases, there was a statistically significant difference in the sudden urge to urinate, accidental loss of a small loss of urine, nocturia, and urine loss associated with a strong desire (P values: 0.02, 0.014, 0.01, and < 0.001, respectively). See supplementary material. Finally, there was a difference between energy drinkers in their wake-up at night due to urination (P value: 0.023). See supplementary material.

On the other hand, there was a statistically significant difference between academic phases, Energy drinks, and coffee drinks for health-related quality of life (HRQL) (P values: 0.002, 0.004, and 0.042, respectively). There was an increase in health-related quality of life in the clinical phase than the academic phase (median [IQR]: 95.38 [83.08, 100], and 92.3 [73.85, 92.31], respectively). Also, Energy drinking was associated with a decrease in quality of life (median [IQR]: 90.77 [73.85, 98.46] and 95.38 [81.54, 100]). Finally, coffee drinking was also associated with a decrease in quality of life (median [IQR]: 93.8[80, 98.46] and 95.38[83.08, 100]).

**Table 3:** Association between sociodemographic data and overactive bladder symptoms bother and its health-related quality of life.

Variable	Arm	*N*	%	Median [IQR]	*P* value
Overactive bladder symptoms bother (OAB)					
Gender	Female	490	48.85%	10[3.33, 23.33]	0.686
	Male	513	51.15%	13.33[3.33, 25]	
Academic phase	Academic phase	259	25.82%	13.33[6.67, 30]	**0.003**
	Clinical phase	744	74.18%	10[3.33, 23.33]	
BMI*	Underweight (BMI < 18.5)	50	4.99%	10[3.33, 20]	0.393
	Normal weight (BMI 18.5–24.9)	664	66.20%	10[3.33, 23.33]	
	Overweight (BMI 25–29.9)	226	22.53%	13.33[3.33, 26.67]	
	Obese (BMI 30–34.9)	44	4.39%	16.67[6.67, 26.67]	
	Morbidly obese (BMI ≥ 35)	19	1.89%	6.67[0, 26.67]	
Smoking	No	954	95.11%	10[3.33, 23.33]	0.965
	Yes	49	4.89%	10[3.33, 23.33]	
Energy drinks	No	837	83.45%	10[3.33, 23.33]	0.086
	Yes	166	16.55%	16.67[3.33, 30]	
Coffee drinks	No	453	45.16%	10[3.33, 23.33]	0.160
	Yes	550	54.84%	13.33[3.33, 23.33]	
Tea drinks	No	326	32.50%	10[3.33, 23.33]	0.532
	Yes	677	67.50%	13.33[3.33, 23.33]	
Health-related quality of life (HRQL)					
Variable	Arm	N	%	Median[IQR]	P value
Gender	Female	490	48.85%	95.38[81.53, 98.46]	0.238
	Male	513	51.15%	93.85[79.23, 98.46]	
Academic phase	Academic phase	259	25.82%	92.3[73.85, 92.31]	**0.002**
	Clinical phase	744	74.18%	95.38[83.08, 100]	
BMI*	Underweight (BMI < 18.5)	50	4.99%	96.62[ 81.15, 100]	0.467
	Normal weight (BMI 18.5–24.9)	664	66.20%	93.85 [81.54, 99.62]	
	Overweight (BMI 25–29.9)	226	22.53%	93.08 [78.46, 98.46]	
	Obese (BMI 30–34.9)	44	4.39%	93.08 [81.92, 98.46]	
	Morbidly obese (BMI ≥ 35)	19	1.89%	92.31 [76.92, 98.46]	
Smoking	No	954	95.11%	93.85[81.15, 98.46]	0.390
	Yes	49	4.89%	92.3[76.15, 98.46]	
Energy drinks	No	837	83.45%	95.38[81.54, 100]	**0.004**
	Yes	166	16.55%	90.77[73.85, 98.46]	
Coffee drinks	No	453	45.16%	95.38[83.08, 100]	**0.042**
	Yes	550	54.84%	93.8[80, 98.46]	
Tea drinks	No	326	32.50%	95.38[81.54, 100]	0.105
	Yes	677	67.50%	93.85[80, 98.46]	

Mann–Whitney U test.

*Kruskal–Wallis test.

### Linear regression

Spearman correlation between overactive bladder symptoms bother, and its related quality of life showed a statistically significant negative correlation (ρ: − 0.708, 95% CI [− 0.738, − 0.674], P value: <0.001). A one-model linear regression showed that an increase in the OAB bother transformed score by one point would decrease the HRQL transformed score by 0.66 points (95% CI [− 0.702, − 0.617], P value: <0.001). This model could explain 47.9% of the relation between OAB symptoms bother and its related quality of life. See supplementary material.

Meanwhile, multiple linear regression was used to assess the relation between HRQL and OAB bother, with sociodemographic factors still showing a statistically significant correlation between HRQL and OAB bother. (95% CI [− 0.698, − 0.611], P value: <0.001). This model explained 48% of this relation. See Table 4.

Additionally, the Academic phase showed a statistically significant association with both OAB and HRQL (p values: 0.001 and 0.019, respectively). The analysis showed that the academic phase had more OAB symptoms bother than the clinical phase. This made the clinical phase have higher quality of life scores than the academic phase. See Table 4.

Interestingly, gender did not show a significant correlation with OAB bother or HRQL, while an increase in age showed a significant decrease in HRQL. (P value: 0.019). Finally, energy drinking was associated with an increase in OAB bother, with no statistically significant decrease in its related quality of life.

**Table 4:** Multiple linear regression assessing the effect of several risk factors on overactive bladder symptoms, bother, and their related quality of life.

Model	Unstandardized coefficients		Standardized coefficients	t	*P* value	95% CI for B
	B	Std. error	β			
Overactive bladder symptoms bother (OAB)						
(Constant)	2.066	8.558		0.241	0.809	(− 14.73, 18.7)
Age	0.541	0.437	0.054	1.238	0.216	(− 0.317, 1.4)
Gender (male–female)	0.836	1.105	0.025	0.757	0.449	(− 1.332, 3)
Academic phase (Clinical-Academic)	− 5.448	1.670	− 0.141	− 3.262	0.001*	(− 8.726, − 2.171)
BMI	0.659	0.741	0.028	0.889	0.374	(− 0.795, 2.112)
Rate your satisfaction with your academic performance	0.466	0.641	0.025	0.726	0.468	(− 0.793, 1.725)
Rate your satisfaction with your social life	1.327	0.650	0.072	2.040	0.042*	(0.051, 2.603)
Rate your stress level	1.259	0.497	0.083	2.535	0.011*	(0.284, 2.234)
smoking	− 2.331	2.576	− 0.030	− 0.905	0.366	(− 7.387, 2.725)
Energy drinks (Yes–No)	3.045	1.528	0.067	1.992	0.047*	(0.046, 6.043)
Coffee (Yes–No)	0.727	1.131	0.021	0.643	0.520	(− 1.492, 2.946)
Tea (Yes–No)	− 0.668	1.168	− 0.018	− 0.572	0.568	(− 2.960, 1.625)
Health-related quality of life (HRQL)						
(Constant)	112.850	5.961		18.932	< 0.001	(101.15, 124.55)
OAB bother	− 0.655	0.022	− 0.687	− 29.582	< 0.001*	(− 0.698, − 0.611)
Age	− 0.714	0.305	− 0.075	− 2.340	0.019*	(− 1.312, − 0.115)
Gender (male–female)	− 0.689	0.770	− 0.021	− 0.895	0.371	(− 2.199, 0.822)
Academic phase (Clinical-Academic)	2.745	1.170	0.075	2.347	0.019*	(0.45, 5.04)
BMI	− 0.013	0.516	− 0.001	− 0.026	0.979	(− 1.026, 0.999)
Rate your satisfaction with your academic performance	0.144	0.447	0.008	0.321	0.748	(− 0.733, 1.021)
Rate your satisfaction with your social life	0.339	0.454	0.019	0.746	0.456	(− 0.552, 1.230)
Rate your stress level	− 0.155	0.347	− 0.011	− 0.447	0.655	(− 0.836, 0.526)
Smoking	− 0.552	1.795	− 0.007	− 0.307	0.759	(− 4.075, 2.971)
Energy drinks (Yes–No)	− 1.072	1.067	− 0.025	− 1.005	0.315	(− 3.165, 1.021)
Coffee (Yes–No)	− 0.188	0.788	− 0.006	− 0.239	0.811	(− 1.734, 1.357)
Tea (Yes–No)	− 0.911	0.814	− 0.026	− 1.119	0.263	(− 2.507, 0.686)

## Discussion

Our study aimed to assess the prevalence of the overactive bladder (OAB) bother among medical students, and its impact on health-related quality of life (HRQL). We found that approximately 15% of students experienced some OAB symptoms, such as being uncomfortable urinating and waking up at night to urinate, which were the most notable symptoms. Despite the low prevalence of OAB reported, we found that about half of them reported a decline in HRQL. Also, we revealed a strong negative association between OAB bother and health-related quality of life (HRQL), as increasing OAB bother score was associated with a reduction in the score of HRQL by about 0.687 (P value: <0.001).

The relatively high frequencies of nocturia (31.2%) and urge urinary incontinence (21.4%) observed in this young population may be influenced by several factors. These include reliance on self-reported symptoms, lifestyle behaviors common among medical students such as high caffeine and energy drink consumption, academic stress, and potential selection bias related to online convenience sampling. Furthermore, in the absence of population-based data on urinary symptoms among young adults in Egypt, it remains unclear whether these findings reflect a true local pattern or are partly attributed to methodological factors. Therefore, these results should be interpreted with caution.

Several studies have reported variable prevalence of OAB. Abuorouq et al. 2024^15^ was a cross-sectional study among the Jordanian medical population. They used the OABSS scale^19^ to diagnose OAB. They reported a high prevalence being 44.5%. Shawahna et al. 2021^16^ was another cross-sectional study among medical students in Palestine. They estimate the prevalence to be 21.6% among medical and dental students. Although the Palestine study showed a similar prevalence as ours, the variation between us and Abuorouq et al., 2024 may be due to different tools, as diagnosing OAB is a subjective diagnosis, making it difficult to accurately diagnose it.

OAB is an age-related disorder with prevalence rising in the middle-aged and older adults^20^. Our study did not show any effect of age on the OAB symptoms bother. This can be explained by the specific age group that we targeted (medical students). This category usually has a similar lifestyle due to the educational burden; however, other studies in the general population have reported different prevalence of OAB. Qudah et al., 2024^14^ was a cross-sectional study among the Jordanian general population. They showed that the odds of being more than 45 years old increase the prevalence of having OAB by about 1.13-fold. Also, Özgür et al. 2012 revealed a higher prevalence of OAB among Turkish midwifery students, being 35.4%^21^. Although we found no effect of age in particular on OAB.

The broad range of 18–27 years included students at different academic stages, which may be associated with varying levels of academic stress. This variability could influence urinary symptoms. So, it was noticed that the OAB symptoms were significantly more frequent in academic years (the first two and a half years in medical schools) students than those in clinical years. In our study, the symptoms such as Urgency (*p* = 0.020), nocturia (*p* = 0.010), urge incontinence (p = < 0.001) were the most significant in the academic year’s students. This was reflected in quality of life, with academic phase students showing a significant reduction of about 0.075 compared to the clinical phase. They revealed that the basic years experienced more OAB symptoms than the clinical years. It aligned with Jordan’s study^15^(p = < 0.001). Academic phase students usually face a much higher stress level than the clinical phase students, especially in Egypt, due to several factors. First, the learning style in the medical field in Egypt is very different than the learning style in school life, leading to putting a high level of stress on them to cope with the medical learning style. The second reason is due to the cultural expectations from the medical students, especially the first-generation medical students, as the parents expect them to know their illness, even when they are still in their academic phase. All these stressful conditions usually lead to an increase in stress-related disorders, including OAB. On the other hand, Shawahna et al. 2012^16^ showed no difference between these two groups. This can be due to the inclusion of dental students in the study.

Additionally, we revealed that no significant difference was observed in gender in OAB or its related quality of life. This finding aligned with findings in Abuorouq et al. 2024^15^(*p* = 0.347), NOBLE study^15^(female, 16.9% vs. male, 16%). Liang et al. 2022^22^ was a cross-sectional study among Chinese university students, revealing a prevalence being 6%. They found a significant difference between females and males, as about 6.7% of the females, while about 4.7% of the males had OAB. This difference between us and theirs may be due to cultural differences or because they included all university students, while we specified our findings to medical students only. Their findings were consistent with previous studies. Such as the EPIC study^23^(female, 12.8% vs. male, 10.8%), EpiLUTS^24^(female, 43.1% vs. male, 27.2), and Milsom^25^(female, 17.4 vs. male, 15.6%).

Furthermore, based on the definition of overactive bladder as the presence of storage symptoms, such as urgency with or without urge incontinence, and associated with frequency or nocturia. In a recent review, Wagg et al. 2024^26^ reported that urgency was the most bothersome symptom in OAB, EpiLUTS^24^ reported that urgency and urge incontinence were the most significant bothersome symptoms (female 15.3% vs. male 9.3%), in Przydacz et al. 2020^13^. reported that the urgency was the most bothersome symptom in men, while the most bothersome symptom in women was urge incontinence. Finally, Mitchell et al. 2014^27^. Approximately 48% of OAB patients reported urgency or urgency incontinence as their most bothersome symptom. In our study, the urgency was found to be widely rated as the most bothersome and significant symptom in females rather than males (*p* = 0.008).

Although previous studies showed that diet had an association with OAB^28^, our results suggest no association between overactive bladder and BMI (P value: 0.374). This may be due to the fact that most students had a normal body mass index (BMI) (about 66.2%), while the obese participants represent only 6.28% of the included participants, which underpowers the results of this association. This was consistent with Abuorouq et al. 2024^15^, and Shawahna et al. 2021^16^, who also reported no association. On the other hand, Hajjar et al. 2022^15^ was a cross-sectional study among nulliparous female university students. They found a statistically significant association between OAB and BMI. Overall, further studies are needed to assess the relation between OAB and obese patients.

Interestingly, Energy drinks significantly increased the OAB bother, while other drinks, such as coffee drinks or tea drinks, showed no effect. Although carbonated drinks such as energy drinks and caffeinated drinks were associated with increasing OAB symptoms^29^, our findings found no difference in either coffee or tea drinks. This may be because all the participants are medical students who usually have a high level of caffeine from coffee or tea daily, while most of them do not usually have energy drinks. Additionally, drinking soft drinks and coffee was associated with a significant reduction in the HRQL in the binary comparison, while in the regression model, they turned into non-significant associations. This variation is because the regression model tries to assess the relationship between variables in consideration of other cofounders in the model, making the results more representative of the real association. Also, the non-association between both drinking soft drinks and a reduction in the quality of life may be due to the nature of the study, as this is a cross-sectional study, which can lead to selection bias in the results. An important note should also be considered, as we did not ask about the usual amount of these drinks because it is a subjective outcome, and every person has their own habit of drinking a usual number of cups of coffee, tea, or energy drinks. Because of this variation, future studies should concentrate on the association between the number of cups of carbonated drinks and OAB prevalence. On the other hand, we also did not find an association between smoking and OAB bother, despite previous studies that suggested an association^15^. Our findings were consistent with those of Shiri et al. 2008^30^, who found no association between smoking (*P* = 0.54), coffee consumption (*P* = 0.09), and nocturia. Despite that, Bradley et al. 2017^31^ found a positive association between tea and nocturia. Additionally, Tikkinen et al. 2009^32^ proved an association between smoking and nocturia, and Hajjar et al. 2022^15^ found that energy drink consumption didn’t associate with nocturia.

Although tobacco use has been reported as a risk factor for increased OAB symptom burden and impaired HRQL in several studies^15,33^. We agreed with them in domain-specific impairment, such as social functioning and daily activity limitation, but our findings suggest that in young medical students, this association is less apparent than the results of studies by Milsom et al. 2012^25^ and Coyne et al., 2004^34^. This may be attributed to the low prevalence of smokers in our study and the young age of participation, which means that the bladder and pelvic floor effects of tobacco are less likely to manifest.

### Strengths and limitations

Our study has several strengths. To our knowledge, this is the first cross-sectional study to be conducted in Egypt to assess the prevalence of OAB bother among medical students and its related quality of life. We assessed the risk factors of this population to identify the modifiable risk factors for these students. Finally, our large sample size enhanced statistical power.

Nevertheless, certain limitations should be acknowledged. The cross-sectional design didn’t prove the causal relation between the exposure and outcome. Data were collected from a self-administered questionnaire that may introduce self -selection bias, particularly for sensitive behaviors such as smoking, and the low prevalence of smoking reduced the ability to detect the potential association with HRQL, as students experiencing urinary symptoms are more likely to participate than asymptomatic individuals, which may substantially overestimate the true prevalence of OAB. The study was confined to medical students, which may limit the generalizability of the findings to other young adults. Also, some sociodemographic questions were yes or no questions, particularly questions about coffee, tea, or soft drinks. The limitation arises because in real life, people may drink them but not regularly, while they regularly prefer one of these drinks over the others. In addition, the use of an OAB-q SF as a diagnostic toll limit the results findings because it is not designed mainly for diagnosing OAB.

## Conclusion

This study was a cross-sectional study among medical students in Egypt to assess the prevalence of overactive bladder bother and its related quality of life. Among 1003 participants, the prevalence of OAB was about 15% with no gender or age difference. We found that about half of the participants reported a decline in health-related quality of life. Additionally, we reported a significant increase in the OAB bother among the academic phase students (the first two and a half years in medical schools) compared to the clinical phase students, with a significant association in their health-related quality of life. Energy drinks were found to have a significant association with the increase in the OAB bother, while coffee and tea drinks did not have an association. Finally, both BMI and smoking had no association with OAB. Future studies should be performed, such as cohort or clinical trials, to further assess these risk factors.

## Supplementary Information

Supplementary Information.

## Data Availability

All data are provided in the manuscript and its related supplementary material.
