# Supplementary material for: Prevalence and risk factors of overactive bladder syndrome among Egyptian medical students, and its impact on health-related quality of life, cross-sectional study
Source: Sci Rep. 2026 May 27;16:16437. doi: 10.1038/s41598-026-53181-4 (PMC13216574; doi:10.1038/s41598-026-53181-4)
Supplement: Supplementary file 1 — Supplementary Information. [file 41598_2026_53181_MOESM1_ESM.docx]

**Supplementary material**

| Sociodemographic data between medical students. | | | |  |
| --- | --- | --- | --- | --- |
|  |  | N | % | |
| Age | mean ±SD | 21.57±1.684 | | |
| Gender | Female | 490 | 48.85% | |
|  | Male | 513 | 51.15% | |
| What is your current academic phase | Academic phase | 259 | 25.82% | |
|  | Clinical phase | 744 | 74.18% | |
| What is your current academic year | First year | 142 | 14.16% | |
|  | Second year | 117 | 11.67% | |
|  | Third year | 229 | 22.83% | |
|  | Fourth year | 148 | 14.76% | |
|  | Fifth year | 367 | 36.59% | |
| What is your body weight | Underweight (BMI <18.5) | 50 | 4.99% | |
|  | Normal weight (BMI 18.5–24.9) | 664 | 66.20% | |
|  | Overweight (BMI 25–29.9) | 226 | 22.53% | |
|  | Obese (BMI 30–34.9) | 44 | 4.39% | |
|  | Morbidly obese (BMI ≥35) | 19 | 1.89% | |
| Do you have a chronic disease? | No | 933 | 93.02% | |
|  | Yes | 70 | 6.98% | |
| Have you had previous surgery? | No | 827 | 82.45% | |
|  | Yes | 176 | 17.55% | |
| Do you have a history of trauma? | No | 910 | 90.73% | |
|  | Yes | 93 | 9.27% | |
| Rate your satisfaction with your academic performance | Very satisfied | 94 | 9.37% | |
|  | satisfied | 450 | 44.87% | |
|  | some-what | 319 | 31.80% | |
|  | unsatisfied | 111 | 11.07% | |
|  | Very unsatisfied | 29 | 2.89% | |
| Rate your satisfaction with your social life | Very satisfied | 124 | 12.36% | |
|  | satisfied | 434 | 43.27% | |
|  | some-what | 314 | 31.31% | |
|  | unsatisfied | 109 | 10.87% | |
|  | Very unsatisfied | 22 | 2.19% | |
| Rate your stress level | 1 | 84 | 8.37% | |
|  | 2 | 115 | 11.47% | |
|  | 3 | 364 | 36.29% | |
|  | 4 | 286 | 28.51% | |
|  | 5 | 154 | 15.35% | |
| Do you have a chronic disease? | No | 933 | 93% | |
|  | Yes | 70 | 7% | |
| Do you have a previous surgery? | No | 827 | 82.5% | |
|  | Yes | 176 | 17.5% | |
| Do you have a history of trauma? | No | 910 | 90.7% | |
|  | Yes | 93 | 9.3% | |
| Are you a smoker? | No | 954 | 95.11% | |
|  | Yes | 49 | 4.89% | |
| Do you usually have energy drinks? | No | 837 | 83.45% | |
|  | Yes | 166 | 16.55% | |
| Do you usually have coffee? | No | 453 | 45.16% | |
|  | Yes | 550 | 54.84% | |
| Do you usually have tea? | No | 326 | 32.50% | |
|  | Yes | 677 | 67.50% | |
| Overactive bladder symptoms transformed score | Median[IQR] | 10[3.33, 23.33] | | |
| HRQL transformed score | Median [IQR] | 93.8[80, 98.46] | | |
| Overactive bladder symptoms bother | No | 850 | 84.70% | |
|  | Yes | 153 | 15.30% | |
| HRQL decrease | No | 485 | 48.35% | |
|  | Yes | 518 | 51.65% | |

| **Overactive Bladder Symptoms bother** | | | | | | |  |
| --- | --- | --- | --- | --- | --- | --- | --- |
| Symptoms | Not at all | A little bit | Some-what | Quite bit | A great deal | A very great deal | |
| An uncomfortable urge to urinate? | 469 (46.8%) | 257(25.6%) | 143 (14.3%) | 81(8.1%) | 37 (3.7%) | 16(0.6%) | |
| A sudden urge to urinate with little or no warning? | 612 (61%) | 200 (19.9%) | 107 (10.7%) | 44 (4.4%) | 27 (2.7%) | 13 (1.3%) | |
| Accidental loss of small amounts of urine? | 718 (71.6%) | 142 (14.2%) | 71 (7.1%) | 39 (3.9%) | 24 (2.4%) | 9 (0.9%) | |
| Nighttime urination? | 594 (59.2%) | 204 (20.3%) | 99 (9.9%) | 52 (5.2%) | 41 (4.1%) | 13 (1.3%) | |
| Waking up at night because you had to urinate? | 373 (37.2%) | 318 (31.7%) | 159 (15.9%) | 74 (7.4%) | 49 (4.9%) | 30 (3%) | |
| Urine loss associated with a strong desire to urinate? | 625 (62.3%) | 154 (15.4%) | 113 (11.3%) | 51 (5.1%) | 48 (4.8%) | 12 (1.2%) | |

| Health-related quality of life (HRQL) | | | | | | |  |
| --- | --- | --- | --- | --- | --- | --- | --- |
| symptoms | None of the time | A little of the time | Some of the time | A good bit of the time | Most of the time | All of the time | |
| Caused you to plan ‘‘escape routes’’ to restrooms in public places? | 626 (62.4%) | 202 (20.1%) | 108 (10.8%) | 35 (3.5%) | 25 (2.5%) | 7 (0.7%) | |
| Made you feel like there is something wrong with you? | 553 (55.1%) | 229 (22.8%) | 125 (12.5%) | 45 (4.5%) | 30 (3%) | 21 (2.1%) | |
| Interfered with your ability to get a good night’s rest? | 567 (56.5%) | 222 (22.1%) | 121 (12.1%) | 43 (4.3%) | 36 (3.6%) | 14 (1.4%) | |
| Made you frustrated or annoyed about the amount of time you spend in the restroom? | 587 (58.5%) | 216 (21.5%) | 115 (11.5%) | 48 (4.8%) | 26 (2.6%) | 11 (1.1%) | |
| Made you avoid activities away from restrooms (i.e., walks, running, hiking)? | 721 (71.9%) | 135 (13.5%) | 81 (8.1%) | 37 (3.7%) | 18 (1.8%) | 11 (1.1%) | |
| Awakened you during sleep? | 469 (46.8%) | 301 (30%) | 125 (12.5%) | 57 (5.7%) | 39 (3.9%) | 12 (1.2%) | |
| Caused you to decrease your physical activities (exercising, sports, etc.)? | 743 (74.%) | 121 (12.1%) | 81 (8.1%) | 28 (2.8%) | 20 (2%) | 10 (1%) | |
| Caused you to have problems with your partner or spouse? | 830 (82.8%) | 77 (7.7%) | 54 (5.4%) | 23 (2.3%) | 11 (1.1%) | 8 (0.8%) | |
| Made you uncomfortable while traveling with others because of needing to stop for a restroom? | 636 (63.4%) | 183 (18.2%) | 105 (10.5%) | 41 (4.1%) | 23 (2.3%) | 15 (1.5%) | |
| Affected your relationships with family and friends? | 793 (79.1%) | 94 (9.4%) | 60 (6%) | 26 (2.6%) | 22 (2.2%) | 8 (0.8%) | |
| Interfered with getting the amount of sleep you needed? | 656 (65.4%) | 172 (17.1%) | 88 (8.8%) | 49 (4.9%) | 21 (2.1%) | 17 (1.7%) | |
| Caused you embarrassment? | 685 (68.3%) | 157 (15.7%) | 97 (9.7%) | 37 (3.7%) | 15 (1.5%) | 12 (1.2%) | |
| Caused you to locate the closest restroom as soon as you arrive at a place you have never been? | 669 (66.7%) | 164 (16.4%) | 102 (12.2%) | 33 (3.3%) | 22 (2.2%) | 13 (1.3%) | |

| **Association between sociodemographic data and overactive bladder symptoms bother and its health-related quality of life** | | | | | |
| --- | --- | --- | --- | --- | --- |
| **Overactive bladder symptoms bother (OAB)** | | | | | |
| Variable | Arm | N | % | Median[IQR] | P value |
| Gender | Female | 490 | 48.85% | 10[3.33, 23.33] | 0.686 |
|  | Male | 513 | 51.15% | 13.33[3.33, 25] |  |
| Academic phase | Academic phase | 259 | 25.82% | 13.33[6.67, 30] | **0.003** |
|  | Clinical phase | 744 | 74.18% | 10[3.33, 23.33] |  |
| BMI* | Underweight (BMI <18.5) | 50 | 4.99% | 10[3.33, 20] | 0.393 |
|  | Normal weight (BMI 18.5–24.9) | 664 | 66.20% | 10[3.33, 23.33] |  |
|  | Overweight (BMI 25–29.9) | 226 | 22.53% | 13.33[3.33, 26.67] |  |
|  | Obese (BMI 30–34.9) | 44 | 4.39% | 16.67[6.67, 26.67] |  |
|  | Morbidly obese (BMI ≥35) | 19 | 1.89% | 6.67[0, 26.67] |  |
| Smoking | No | 954 | 95.11% | 10[3.33, 23.33] | 0.965 |
|  | Yes | 49 | 4.89% | 10[3.33, 23.33] |  |
| Energy drinks | No | 837 | 83.45% | 10[3.33, 23.33] | 0.086 |
|  | Yes | 166 | 16.55% | 16.67[3.33, 30] |  |
| Coffee drinks | No | 453 | 45.16% | 10[3.33, 23.33] | 0.160 |
|  | Yes | 550 | 54.84% | 13.33[3.33, 23.33] |  |
| Tea drinks | No | 326 | 32.50% | 10[3.33, 23.33] | 0.532 |
|  | Yes | 677 | 67.50% | 13.33[3.33, 23.33] |  |
| **Health-related quality of life (HRQL)** | | | | | |
| Variable | Arm | N | % | Median[IQR] | P value |
| Gender | Female | 490 | 48.85% | 95.38[81.53, 98.46] | 0.238 |
|  | Male | 513 | 51.15% | 93.85[79.23, 98.46] |  |
| Academic phase | Academic phase | 259 | 25.82% | 92.3[73.85, 92.31] | **0.002** |
|  | Clinical phase | 744 | 74.18% | 95.38[83.08, 100] |  |
| BMI* | Underweight (BMI <18.5) | 50 | 4.99% | 96.62[ 81.15, 100] | 0.467 |
|  | Normal weight (BMI 18.5–24.9) | 664 | 66.20% | 93.85 [81.54, 99.62] |  |
|  | Overweight (BMI 25–29.9) | 226 | 22.53% | 93.08 [78.46, 98.46] |  |
|  | Obese (BMI 30–34.9) | 44 | 4.39% | 93.08 [81.92, 98.46] |  |
|  | Morbidly obese (BMI ≥35) | 19 | 1.89% | 92.31 [76.92, 98.46] |  |
| Smoking | No | 954 | 95.11% | 93.85[81.15, 98.46] | 0.390 |
|  | Yes | 49 | 4.89% | 92.3[76.15, 98.46] |  |
| Energy drinks | No | 837 | 83.45% | 95.38[81.54, 100] | **0.004** |
|  | Yes | 166 | 16.55% | 90.77[73.85, 98.46] |  |
| Coffee drinks | No | 453 | 45.16% | 95.38[83.08, 100] | **0.042** |
|  | Yes | 550 | 54.84% | 93.8[80, 98.46] |  |
| Tea drinks | No | 326 | 32.50% | 95.38[81.54, 100] | 0.105 |
|  | Yes | 677 | 67.50% | 93.85[80, 98.46] |  |
| Mann-Whitney U Test  * Kruskal Wallis Test | | | | | |

| **Difference between genders in OAB and HRQL in each study phase** | | | | |
| --- | --- | --- | --- | --- |
| What is your current academic phase? | | | N | P value |
| Academic phase | OAB | Female | 124 | 0.796 |
|  |  | Male | 135 |  |
|  |  | Total | 259 |  |
|  | HRQL | Female | 124 | 0.761 |
|  |  | Male | 135 |  |
|  |  | Total | 259 |  |
| Clinical phase | OAB | Female | 366 | 0.516 |
|  |  | Male | 378 |  |
|  |  | Total | 744 |  |
|  | HRQL | Female | 366 | 0.254 |
|  |  | Male | 378 |  |
|  |  | Total | 744 |  |
| **Mann-Whitney U Test** | | | | |

| The difference between genders on each OAB symptom | | | | | | | | |
| --- | --- | --- | --- | --- | --- | --- | --- | --- |
| Symptoms | | Not at all | A little bit | Some-what | Quite bit | A great deal | A very great deal | P value |
| An uncomfortable urge to urinate? | Female | 229(46.7% | 133( 27.1%) | 66 (13.5%) | 36 (7.3%) | 17 (3.5%) | 9 (1.8%) | 0.680 |
|  | Male | 240(46.8%) | 124(24.2%) | 77 (15%) | 45 (8.8%) | 20 (3.9%) | 7 (1.4%) |  |
| A sudden urge to urinate with little or no warning? | Female | 322 (65.7%) | 82 (16.7%) | 47 (9.6%) | 20 (4.1%) | 13 (2.7%) | 6 (1.2%) | **0.008** |
|  | Male | 290 (56.5%) | 118 (23%) | 60 (11.7%) | 24 (4.7%) | 14 (2.7%) | 7 (1.4%) |  |
| Accidental loss of small amounts of urine? | Female | 345 (70.4%) | 78 (15.9%) | 31 (6.3%) | 20 (4.1%) | 12 (2.4%) | 4 (0.8%) | 0.544 |
|  | Male | 373 (72.7%) | 64 (15.5%) | 40 (3.7%) | 19 (3.7%) | 12 (2.3%) | 5 (1%) |  |
| Nighttime urination? | Female | 298 (60.8%) | 98 (20%) | 49 (10%) | 14 (2.9%) | 23 (4.7%) | 8 (1.6%) | 0.289 |
|  | Male | 296 (57.7%) | 106 (20.7%) | 50 (9.7%) | 38 (7.4%) | 18 (3.5%) | 5 (1%) |  |
| Waking up at night because you had to urinate? | Female | 177 (36.1%) | 163 (33.3%) | 80 (16.3%) | 29 (5.9%) | 27 (5.5%) | 14 (2.9%) | 0.856 |
|  | Male | 196 (38.2%) | 155 (30.2%) | 79 (15.4%) | 45 (8.8%) | 22 (4.3%) | 16 (3.1%) |  |
| Urine loss associated with a strong desire to urinate? | Female | 303 (61.8%) | 75 (15.3%) | 57 (11.6%) | 19 (3.9%) | 27 (5.5%) | 9 (1.8%) | 0.662 |
|  | Male | 322 (62.8%) | 79 (15.4%) | 56 (10.9%) | 32 (6.2%) | 21 (4.1%) | 3 (0.6%) |  |
| Mann-Whitney U Test | | | | | | | | |

| Difference between the Academic phases on each OAB symptom | | | | | | | | | | | | | | | | |
| --- | --- | --- | --- | --- | --- | --- | --- | --- | --- | --- | --- | --- | --- | --- | --- | --- |
| Symptoms | | | Not at all | | A little bit | | Some-what | | Quite bit | | A great deal | | A very great deal | | P value | |
| An uncomfortable urge to urinate? | Academic phase | 128 (49.4%) | | 63 (24.3%) | | 31 (12%) | | 22 (8.5%) | | 11 (4.2%) | | 4 (1.5%) | | 0.446 | |  |
|  | Clinical phase | 341 (45.8%) | | 194 (26.1%) | | 112 (15.1%) | | 59 (7.9%) | | 26 (3.5%) | | 12 (1.6%) | |  |  |  |
| A sudden urge to urinate with little or no warning? | Academic phase | 144 (55.6%) | | 53 (20.5%) | | 35 (13.5%) | | 14 (5.4%) | | 8 (3.1%) | | 5 (1.9%) | | **0.020** | |  |
|  | Clinical phase | 468 (62.9%) | | 147 (19.8%) | | 72 (9.7%) | | 30 (4%) | | 19 (2.6%) | | 8 (1.1%) | |  |  |  |
| Accidental loss of small amounts of urine? | Academic phase | 171 (66%) | | 44 (17%) | | 18 (6.6%) | | 6 (2.4%) | | 13 (5%) | | 7 (2.7%) | | **0.014** | |  |
|  | Clinical phase | 547 (73.5%) | | 98 (13.2%) | | 53 (7.1%) | | 33 (4.4%) | | 11 (1.5%) | | 2 (0.3%) | |  |  |  |
| Nighttime urination? | Academic phase | 139 (53.7%) | | 51 (19.7%) | | 32 (12.4%) | | 17 (6.6%) | | 17 (6.6%) | | 3 (1.2%) | | **0.010** | |  |
|  | Clinical phase | 455 (61.2%) | | 153 (20.6%) | | 67 (9%) | | 35 (4.7%) | | 24 (3.2%) | | 10 (1.3%) | |  |  |  |
| Waking up at night because you had to urinate? | Academic phase | 96 (37.1%) | | 66 (25.5%) | | 47 (18.1%) | | 21 (8.1%) | | 19 (7.3%) | | 10 (3.9%) | | 0.126 | |  |
|  | Clinical phase | 277 (37.2%) | | 252 (33.9%) | | 112 (15.1%) | | 53 (7.1%) | | 30 (4%) | | 20 (2.7%) | |  |  |  |
| Urine loss associated with a strong desire to urinate? | Academic phase | 139 (53.7%) | | 40 (15.4%) | | 31 (12%) | | 18 (6.9%) | | 25 (9.7%) | | 6 (2.3%) | | **<0.001** | |  |
|  | Clinical phase | 486 (65.3%) | | 114 (15.%) | | 82 (11%) | | 33 (4.4%) | | 23 (3.1%) | | 6 (0.8%) | |  |  |  |
| Mann-Whitney U Test | | | | | | | | | | | | | | | |  |

| **Difference between genders in each OAB symptom in each study phase** | | | | |
| --- | --- | --- | --- | --- |
| What is your current academic phase? | | | N | P value |
| Academic phase | An uncomfortable urge to urinate? | Female | 124 | 0.984 |
|  |  | Male | 135 |  |
|  |  | Total | 259 |  |
|  | A sudden urge to urinate with little or no warning? | Female | 124 | 0.137 |
|  |  | Male | 135 |  |
|  |  | Total | 259 |  |
|  | Accidental loss of small amounts of urine? | Female | 124 | 0.780 |
|  |  | Male | 135 |  |
|  |  | Total | 259 |  |
|  | Nighttime urination? | Female | 124 | 0.835 |
|  |  | Male | 135 |  |
|  |  | Total | 259 |  |
|  | Waking up at night because you had to urinate? | Female | 124 | 0.737 |
|  |  | Male | 135 |  |
|  |  | Total | 259 |  |
|  | Urine loss associated with a strong desire to urinate? | Female | 124 | 0.759 |
|  |  | Male | 135 |  |
|  |  | Total | 259 |  |
| Clinical phase | An uncomfortable urge to urinate? | Female | 366 | 0.616 |
|  |  | Male | 378 |  |
|  |  | Total | 744 |  |
|  | A sudden urge to urinate with little or no warning? | Female | 366 | **0.031** |
|  |  | Male | 378 |  |
|  |  | Total | 744 |  |
|  | Accidental loss of small amounts of urine? | Female | 366 | 0.565 |
|  |  | Male | 378 |  |
|  |  | Total | 744 |  |
|  | Nighttime urination? | Female | 366 | 0.264 |
|  |  | Male | 378 |  |
|  |  | Total | 744 |  |
|  | Waking up at night because you had to urinate? | Female | 366 | 0.985 |
|  |  | Male | 378 |  |
|  |  | Total | 744 |  |
|  | Urine loss associated with a strong desire to urinate? | Female | 366 | 0.678 |
|  |  | Male | 378 |  |
|  |  | Total | 744 |  |
| **Mann-Whitney U Test** | | | | |

| Difference between current smoking status on each OAB symptom | | | | | | | | |
| --- | --- | --- | --- | --- | --- | --- | --- | --- |
| Symptoms | | Not at all | A little bit | Some-what | Quite bit | A great deal | A very great deal | P value |
| An uncomfortable urge to urinate? | No | 441 (46.2%) | 248 (26%) | 137 (14.4%) | 75 (7.9%) | 37 (3.9%) | 16 (1.7%) | 0.197 |
|  | Yes | 28 (57.1%) | 9 (18.4%) | 6 (12.2%) | 6 (12.2%) |  |  |  |
| A sudden urge to urinate with little or no warning? | No | 579 (60.7%) | 190 (19.9%) | 103 (10.8%) | 42 (4.4%) | 27 (2.8%) | 13 (1.4%) | 0.244 |
|  | Yes | 33 (67.3%) | 10 (20.4%) | 4 (8.2%) | 2 (4.1%) |  |  |  |
| Accidental loss of small amounts of urine? | No | 688 (72.1%) | 131 (13.7%) | 66 (6.9%) | 36 (3.8%) | 24 (2.5%) | 9 (0.9%) | 0.160 |
|  | Yes | 30 (61.2%) | 11 (10.2%) | 5 (10.2%) | 3 (6.1%) |  |  |  |
| Nighttime urination? | No | 563 (59%) | 197 (9.7%) | 93 (9.7%) | 50 (5.2%) | 40 (4.2%) | 11 (1.2%) | 0.731 |
|  | Yes | 31 (63.3%) | 7 (12.2%) | 6 (12.2%) | 2 (4.1%) | 1 (2%) | 2 (4.1%) |  |
| Waking up at night because you had to urinate? | No | 354 (37.1%) | 304 (31.9%) | 153 (16%) | 68 (7.1%) | 46 (4.8%) | 29 (29%) | 0.917 |
|  | Yes | 19 (38.8%) | 14 (28.6%) | 6 (12.2%) | 6 (12.2%) | 3 (6.1%) | 1 (2) |  |
| Urine loss associated with a strong desire to urinate? | No | 600 (62.9%) | 142 (14.9%) | 109 (11.4%) | 45 (4.7%) | 46 (4.8%) | 12 (1.3%) | 0.154 |
|  | Yes | 25 (51%) | 12 (24.5%) | 4 (8.2%) | 6 (12.2%) | 2 (4.1%) |  |  |
| Mann-Whitney U Test | | | | | | | | |

| Difference between Energy drinks on each OAB symptoms | | | | | | | | | |
| --- | --- | --- | --- | --- | --- | --- | --- | --- | --- |
| Symptoms | | Not at all | A little bit | Some-what | Quite bit | A great deal | A very great deal | P value |  |
| An uncomfortable urge to urinate? | No | 385 (46%) | 218 (26%) | 124 (14.8%) | 67 (8%) | 33 (3.9%) | 10 (1.2%) | 0.430 |  |
|  | Yes | 84 (50.6%) | 39 (23.5%) | 19 (11.4%) | 14 (8.4%)\ | 4 (8.4%) | 6 (3.6%) |  |  |
| A sudden urge to urinate with little or no warning? | No | 516 (61.6%) | 170 (*20.3%) | 88 (10.5%) | 36 (4.3%) | 18 (2.2%) | 9 (1.1%) | 0.155 |  |
|  | Yes | 96 (57.8%) | 30 (18.1%) | 19 (11.4%) | 8 (4.8%) | 9 (5.4%) | 4 (2.4%) |  |  |
| Accidental loss of small amounts of urine? | No | 605 (72.3%) | 117 (14%) | 61 (7.3%) | 28 (3.3%) | 20 (2.4%) | 6 (0.7%) | 0.212 |  |
|  | Yes | 113 (68.1%) | 25 (15.1%) | 10 (6%) | 11 (6.6%) | 4 (2.4%) | 3 (1.8%) |  |  |
| Nighttime urination? | No | 503 (60.1%) | 178 (21.3%) | 71 (8.5%) | 41 (4.9%) | 36 (4.3%) | 8 (1%) | 0.064 |  |
|  | Yes | 91 (54.8%) | 26 (15.7%) | 28 (16.9%) | 11 (6.6%) | 5 (3%) | 5 (3%) |  |  |
| Waking up at night because you had to urinate? | No | 319 (38.1%) | 273 (32.6%) | 125 (14.9%) | 56 (6.7%) | 41 (4.9%) | 23 (2.7%) | **0.023** |  |
|  | Yes | 54 (32.5%) | 45 (27.1%) | 34 (20.5%) | 18 (10.8%) | 8 (4.8%) | 7 (4.2%) |  |  |
| Urine loss associated with a strong desire to urinate? | No | 529 (63.2%) | 133 (15.9%) | 89 (10.6%) | 40 (4.8%) | 37 (4.4%) | 9 (1.1%) | 0.075 |  |
|  | Yes | 96 (57.8%) | 21 (12.7%) | 24 (14.5%) | 11 (6.6%) | 11 (6.6%) | 3 (1.8%) |  |  |
| Mann-Whitney U Test | | | | | | | | |  |

| Difference between Coffee drinks on each OAB symptoms | | | | | | | | | |
| --- | --- | --- | --- | --- | --- | --- | --- | --- | --- |
| Symptoms | | Not at all | A little bit | Some-what | Quite bit | A great deal | A very great deal | P value |  |
| An uncomfortable urge to urinate? | No | 215 (47.5%) | 116 (25.6%) | 63 (13.9%) | 34 (7.5%) | 21 (4.6%) | 4 (0.9%) | 0.645 |  |
|  | Yes | 254 (46.2%) | 141 (25.6%) | 80 (14.5%) | 47 (8.5%) | 16 (2.9%) | 12 (2.2%) |  |  |
| A sudden urge to urinate with little or no warning? | No | 279 (61.6%) | 94 (20.8%) | 47 (10.4%) | 20 (4.4%) | 7 (1.5%) | 6 (1.3%) | 0.520 |  |
|  | Yes | 333 ((60.5%) | 106 (19.3%) | 60 (10.9%) | 24 (4.4%) | 20 (3.6%) | 7 (1.3%) |  |  |
| Accidental loss of small amounts of urine? | No | 328 (72.4%) | 60 (13.2%) | 35 (7.7%) | 14 (3.1%) | 13 (2.9%) | 3 (0.7%) | 0.644 |  |
|  | Yes | 390 (70.9%) | 82 (14.9%) | 36 (6.5%) | 25 (4.5%) | 11 (2%) | 6 (1.1%) |  |  |
| Nighttime urination? | No | 9 (60%) | 92 (20.3%) | 39 (8.6%) | 25 (5.5%) | 19 (4.2%) | 6 (1.3%) | 0.649 |  |
|  | Yes | 322 (58.5%) | 112 (20.3%) | 60 (10.9%) | 27 (4.9%) | 22 (4%) | 7 (1.3%) |  |  |
| Waking up at night because you had to urinate? | No | 180 (39.7%) | 145 (32%) | 68 (15%) | 25 (5.5%) | 20 (4.4%) | 15 (3.3%) | 0.063 |  |
|  | Yes | 193 (35.1%) | 173 (31.5%) | 91 (16.5%) | 49 (8.9%) | 29 (5.3%) | 15 (2.7%) |  |  |
| Urine loss associated with a strong desire to urinate? | No | 292 (64.5%) | 65 (14.3%) | 51 (11.3%) | 20 (4.4%) | 21 (4.6%) | 4 (0.9%) | 0.203 |  |
|  | Yes | 333 (60.5%) | 89 (16.2%) | 62 (11.3%)' | 31 (5.6%) | 27 (4.9%) | 8 (1.5%) |  |  |
| Mann-Whitney U Test | | | | | | | | |  |

| Difference between Tea drinks on each OAB symptoms | | | | | | | | | |
| --- | --- | --- | --- | --- | --- | --- | --- | --- | --- |
| Symptoms | | Not at all | A little bit | Some-what | Quite bit | A great deal | A very great deal | P value |  |
| An uncomfortable urge to urinate? | No | 147 (45.1%) | 77 (23.6%) | 47 (14.4%) | 32 (9.8%) | 17 (5.2%) | 6 (1.8%) | 0.135 |  |
|  | Yes | 322 (47.6%) | 180 (26.6%) | 96 (14.2%) | 49 (7.2%) | 20 (3%) | 10 (1.5%) |  |  |
| A sudden urge to urinate with little or no warning? | No | 209 (64.1%) | 56 (17.2%) | 27 (8.3%) | 19 (5.8%) | 9 (2.8%) | 6 (1.8%) | 0.331 |  |
|  | Yes | 403 (59.5%) | 144 (21.3%) | 80 (11.8%) | 25 (3.7%) | 18 (2.7%) | 7 (1%) |  |  |
| Accidental loss of small amounts of urine? | No | 238 (73%) | 41 (12.6%) | 20 (6.1%) | 15 (4.6%) | 9 (2.8%) | 3 (0.9%) | 0.619 |  |
|  | Yes | 480 (70.9%) | 101 (14.69%) | 51 (7.5%) | 24 (3.5%) | 15 (2.2%) | 6 (0.9%) |  |  |
| Nighttime urination? | No | 196 (60.1%) | 58 (17.8%) | 26 (8%) | 24 (7.4%) | 16 (4.9%) | 6 (1.8%) | 0.840 |  |
|  | Yes | 398 (58.8%) | 146 (21.6%) | 73 (10.8%) | 28 (4.1%) | 25 (3.7%) | 7 (1%) |  |  |
| Waking up at night because you had to urinate? | No | 139 (42.6%) | 86 (26.4%) | 52 (16%) | 23 (7.1%) | 17 (5.2%) | 9 (2.8%) | 0.123 |  |
|  | Yes | 234 (34.6%) | 232 (34.3%) | 107 (15.8%) | 51 (7.5%) | 32 (4.7%) | 21 (3.1%) |  |  |
| Urine loss associated with a strong desire to urinate? | No | 214 (65.6%) | 45 (13.8%) | 35 (10.7%) | 9 (2.8%) | 17 (5.2%) | 6 (1.8%) | 0.171 |  |
|  | Yes | 411 (60.7%) | 109 (16.1%) | 78 (11.5%) | 42 (6.2%) | 31 (4.6%) | 6 (0.9%) |  |  |
| Mann-Whitney U Test | | | | | | | | |  |

| Difference between Genders in HRQL | | | | | | | | | |
| --- | --- | --- | --- | --- | --- | --- | --- | --- | --- |
| symptoms | | None of the time | A little of the time | Some of the time | A good bit of the time | Most of the time | All of the time | P value |  |
| Caused you to plan ‘‘escape routes’’ to restrooms in public places? | Female | 334 (68.2%) | 86 (17.6%) | 44 (9%) | 12 (2.4%) | 10 (2.4%) | 4 (0.8%) | **<0.001** |  |
|  | Male | 292 (56.9%) | 116 (22.6%) | 64 (12.5%) | 23 (4.5%) | 15 (2.9%) | 3 (0.6%) |  |  |
| Made you feel like there is something wrong with you? | Female | 269 (56.9%) | 112 (22.9%) | 59 (12%) | 20 (4.1%) | 19 (3.9%) | 11 (2.2%) | 0.789 |  |
|  | Male | 284 (55.4%) | 117 (22.8%) | 66 (12.9%) | 25 (4.9%) | 11 (2.1%) | 10 (1.9%) |  |  |
| Interfered with your ability to get a good night’s rest? | Female | 286 (58.4%) | 108 (22%) | 55 (12.2%) | 17 (11.2%) | 16 (3.5%) | 8 (1.6%) | 0.192 |  |
|  | Male | 281 (54.8%) | 114 (22.2%) | 66 (12.9%) | 26 (12.9%) | 20 (5.1%) | 6 (1.2%) |  |  |
| Made you frustrated or annoyed about the amount of time you spend in the restroom? | Female | 291 (59.4%) | 113 (23.1%) | 50 (10.2%) | 19 (3.9%) | 11 (2.2%) | 6 (1.2%) | 0.312 |  |
|  | Male | 296 (57.7%) | 103 (20.1%) | 65 (12.7%) | 29 (5.7%) | 15 (2.9%) | 5 (1%) |  |  |
| Made you avoid activities away from restrooms (i.e., walks, running, hiking)? | Female | 368 (75.1%) | 55 (11.2%) | 36 (7.3%) | 15 (3.1%) | 8 (1.6%) | 8 (1.6%) | **0.046** |  |
|  | Male | 353 (68.8%) | 80 (15.6%) | 45 (8.8%) | 22 (4.3%) | 10 (1.9%) | 3 (0.6%) |  |  |
| Awakened you during sleep? | Female | 237 (48.4%) | 142 (29%) | 58 (11.8%) | 27 (5.5%) | 22 (4.5%) | 4 (0.8%) | 0.398 |  |
|  | Male | 232 (45.2%) | 159 (31%) | 67 (13.1%) | 30 (5.8%) | 17 (3.3%) | 8 (1.6%) |  |  |
| Caused you to decrease your physical activities (exercising, sports, etc.)? | Female | 373 (76.1%) | 63 (12.9%) | 29 (5.9%) | 12 (2.4%) | 8 (1.6%) | 5 (1%) | 0.091 |  |
|  | Male | 370 (72.1%) | 58 (11.3%) | 52 (10.1%) | 16 (3.1%) | 12 (2.3%) | 5 (1%) |  |  |
| Caused you to have problems with your partner or spouse? | Female | 417 (85.1%) | 34 (6.9%) | 19 (3.9%) | 11 (2.2%) | 3 (0.6%) | 6 (1.2%) | 0.057 |  |
|  | Male | 413 (80.5%) | 43 (8.4%) | 35 (6.8%) | 12 (2.3%) | 8 (1.6%) | 2 (0.4%) |  |  |
| Made you uncomfortable while traveling with others because of needing to stop for a restroom? | Female | 312 (63.7%) | 95 (19.4%) | 43 (8.8%) | 19 (3.9%) | 12 (2.4%) | 9 (1.8%) | 0.740 |  |
|  | Male | 324 (63.2%) | 88 (17.2%) | 62 (12.1%) | 22 (4.3%) | 11 (2.1%) | 6 (10.1%) |  |  |
| Affected your relationships with family and friends? | Female | 404 (82.4%) | 36 (7.3%) | 22 (4.5%) | 10 (2%) | 13 (2.7%) | 5 (1%) | **0.017** |  |
|  | Male | 389 (75.8%) | 58 (11.3%) | 38 (7.4%) | 16 (3.1%) | 9 (1.8%) | 3 (0.6%) |  |  |
| Interfered with getting the amount of sleep you needed? | Female | 334 (68.2%) | 81 (16.5%) | 35 (7.1%) | 23 (4.7%) | 9 (1.8%) | 8 (1.6%) | 0.063 |  |
|  | Male | 322 (62.8%) | 91 (17.7%) | 53 (10.3%) | 26 (5.1%) | 12 (2.3%) | 5 (1.8%) |  |  |
| Caused you embarrassment? | Female | 343 (70%) | 75 (15.3%) | 43 (8.8%) | 15 (3.1%) | 6 (1.2%) | 8 (1.6%) | 0.237 |  |
|  | Male | 342 (66.7%) | 82 (10.5%) | 54 (10.5%) | 22 (4.3%) | 9 (1.8%) | 4 (0.8%) |  |  |
| Caused you to locate the closest restroom as soon as you arrive at a place you have never been? | Female | 341 (69.6%) | 75 (8%) | 39 (8%) | 12 (2.4%) | 13 (2.7%) | 10 (2%) | 0.078 |  |
|  | Male | 328 (63.9%) | 89 (12.3%) | 63 (12.3%) | 21 (4.1%) | 9 (1.8%) | 3 (0.6%) |  |  |
| Mann-Whitney U Test | | | | | | | | |  |

| Difference between the Academic phase in HRQL | | | | | | | | | |
| --- | --- | --- | --- | --- | --- | --- | --- | --- | --- |
| symptoms | | None of the time | A little of the time | Some of the time | A good bit of the time | Most of the time | All of the time | P value |  |
| Caused you to plan ‘‘escape routes’’ to restrooms in public places? | Academic phase | 155 (59.8%) | 57 (22%) | 24 (9.3%) | 12 (4.6% | 8 (3.1%) | 3 (1.2%) | 0.308 |  |
|  | Clinical phase | 471 (63.3%) | 145 (19.5%) | 84 (11.3%) | 23 (3.1%) | 17 (2.3%) | 4 (0.5%) |  |  |
| Made you feel like there is something wrong with you? | Academic phase | 130 (50.2%) | 60 (23.2%) | 37 (14.3%) | 10 (3.9%) | 12 (4.6%) | 10 (3.9%) | **0.024** |  |
|  | Clinical phase | 423 (56.9%) | 169 (22.7%) | 88 (11.8%) | 35 (4.7%) | 18 (2.4%) | 11 (1.5%) |  |  |
| Interfered with your ability to get a good night’s rest? | Academic phase | 127 (49%) | 64 (24.2%) | 36 (13.9%) | 15 (5.8%) | 12 (4.6%) | 5 (1.9%) | **0.003** |  |
|  | Clinical phase | 440 (59.1%) | 158 (21.2%) | 85 (11.4%) | 28 (3.8%) | 24(3.2%) | 9 (1.2%) |  |  |
| Made you frustrated or annoyed about the amount of time you spend in the restroom? | Academic phase | 142 (54.8%) | 54 (20.8%) | 37 (14.3%) | 17 (6.6%) | 7 (2.7%) | 2 (0.8%) | 0.091 |  |
|  | Clinical phase | 445 (59.8%) | 162 (21.8%) | 78 (10.45%) | 31 (4.2%) | 19 (2.6%) | 9 (1.2%) |  |  |
| Made you avoid activities away from restrooms (i.e., walks, running, hiking)? | Academic phase | 174 (67.2%) | 37 (14.8%) | 25 (9.7%) | 14 (5.4%) | 5 (1.9%) | 4 (1.5%) | **0.035** |  |
|  | Clinical phase | 547 (73.5%) | 98 (13.2%) | 56 (7.5%) | 23 (3.1%) | 13 (1.7%) | 7 (0.9%) |  |  |
| Awakened you during sleep? | Academic phase | 108 (41.7%) | 75 (29%) | 38 (14.7%) | 19 (7.3%) | 16 (6.2%) | 3 (1.2%) | **0.010** |  |
|  | Clinical phase | 361 (48.5%) | 226 (30.4%) | 87 (11.7%) | 38 (5.1%) | 23 (3.1%) | 9 (1.2%) |  |  |
| Caused you to decrease your physical activities (exercising, sports, etc.)? | Academic phase | 178 (68.7%) | 35 (13.5%) | 25 (9.7%) | 8 (3.1%) | 9 (3.5%) | 4 (1.5%) | **0.015** |  |
|  | Clinical phase | 565 (75.9%) | 86 (11.6%) | 56 (7.5%) | 20 (2.7%) | 11 (1.5%) | 6 (0.8%) |  |  |
| Caused you to have problems with your partner or spouse? | Academic phase | 200 (77.2%) | 26 (10%) | 19 (7.3%) | 7 (2.7%) | 4 (1.5%) | 3 (1.2%) | **0.006** |  |
|  | Clinical phase | 630 (84.7%) | 51 (6.9%) | 35 (4.7%) | 16 (2.2%) | 7 (0.9%) | 5 (0.7%) |  |  |
| Made you uncomfortable while traveling with others because of needing to stop for a restroom? | Academic phase | 156 (60.2%) | 45 (17.4%) | 32 (12.4%) | 13 (5%) | 7 (2.7%) | 6 (2.3%) | 0.115 |  |
|  | Clinical phase | 480 (64.5%) | 138 (18.5%) | 73 (9.8%) | 28 (3.8%) | 16 (2.2%) | 9 (1.2%) |  |  |
| Affected your relationships with family and friends? | Academic phase | 191 (73.7%) | 26 (10%) | 21 (8.1%) | 7 (2.7%) | 10 (3.9%) | 4 (1.5%) | **0.009** |  |
|  | Clinical phase | 602 (80.9%) | 68 (9.1%) | 39 (5.2%) | 19 (2.6%) | 12 (1.6%) | 4 (0.5%) |  |  |
| Interfered with getting the amount of sleep you needed? | Academic phase | 157 (60.6%) | 43 (16.6%) | 27 (10.4%) | 18 (6.9%) | 8 (3.1%) | 6 (2.3%) | **0.022** |  |
|  | Clinical phase | 499 (67.1%) | 129 (17.3%) | 61 (8.2%) | 31 (4.2%) | 13 (1.7%) | 11 (1.5%) |  |  |
| Caused you embarrassment? | Academic phase | 157 (60.6%) | 47 (18.1%) | 30 (11 | 14 (5.4%) | 5 (1.9%) | 6 (2.3%) | **0.001** |  |
|  | Clinical phase | 528 (71%) | 110 (14.8%) | 6 (11.6%) | 23 (3.1%) | 10 (1.3%) | 6 (0.8%) |  |  |
| Caused you to locate the closest restroom as soon as you arrive at a place you have never been? | Academic phase | 156 (60.2%) | 53 (20.5%) | 28 (10.8%) | 11 (4.2%) | 7 (2.7%) | 4 (1.5%) | **0.015** |  |
|  | Clinical phase | 513 (69%) | 111 (14.9%) | 74 (9.9%) | 22 (3%) | 15 2%) | 9 (1.2%) |  |  |
| Mann-Whitney U Test | | | | | | | | |  |

| **Difference between genders in each HRQL in each study phase** | | | | | |
| --- | --- | --- | --- | --- | --- |
| What is your current academic phase? | | | N | P value | |
| Academic phase | Caused you to plan ‘‘escape routes’’ to restrooms in public places? | Female | 124 | **0.011** |  |
|  |  | Male | 135 |  |  |
|  |  | Total | 259 |  |  |
|  | Made you feel like there is something wrong with you? | Female | 124 | 0.561 |  |
|  |  | Male | 135 |  |  |
|  |  | Total | 259 |  |  |
|  | Interfered with your ability to get a good night’s rest? | Female | 124 | 0.700 |  |
|  |  | Male | 135 |  |  |
|  |  | Total | 259 |  |  |
|  | Made you frustrated or annoyed about the amount of time you spend in the restroom? | Female | 124 | 0.694 |  |
|  |  | Male | 135 |  |  |
|  |  | Total | 259 |  |  |
|  | Made you avoid activities away from restrooms (i.e., walks, running, hiking)? | Female | 124 | **0.045** |  |
|  |  | Male | 135 |  |  |
|  |  | Total | 259 |  |  |
|  | Awakened you during sleep? | Female | 124 | 0.556 |  |
|  |  | Male | 135 |  |  |
|  |  | Total | 259 |  |  |
|  | Caused you to decrease your physical activities (exercising, sports, etc.)? | Female | 124 | 0.193 |  |
|  |  | Male | 135 |  |  |
|  |  | Total | 259 |  |  |
|  | Caused you to have problems with your partner or spouse? | Female | 124 | 0.197 |  |
|  |  | Male | 135 |  |  |
|  |  | Total | 259 |  |  |
|  | Made you uncomfortable while traveling with others because of needing to stop for a restroom? | Female | 124 | 0.734 |  |
|  |  | Male | 135 |  |  |
|  |  | Total | 259 |  |  |
|  | Affected your relationships with family and friends? | Female | 124 | 0.532 |  |
|  |  | Male | 135 |  |  |
|  |  | Total | 259 |  |  |
|  | Interfered with getting the amount of sleep you needed? | Female | 124 | 0.823 |  |
|  |  | Male | 135 |  |  |
|  |  | Total | 259 |  |  |
|  | Caused you embarrassment? | Female | 124 | 0.927 |  |
|  |  | Male | 135 |  |  |
|  |  | Total | 259 |  |  |
|  | Caused you to locate the closest restroom as soon as you arrive at a place you have never been? | Female | 124 | 0.622 |  |
|  |  | Male | 135 |  |  |
|  |  | Total | 259 |  |  |
| Clinical phase | Caused you to plan ‘‘escape routes’’ to restrooms in public places? | Female | 366 | **0.006** |  |
|  |  | Male | 378 |  |  |
|  |  | Total | 744 |  |  |
|  | Made you feel like there is something wrong with you? | Female | 366 | 0.993 |  |
|  |  | Male | 378 |  |  |
|  |  | Total | 744 |  |  |
|  | Interfered with your ability to get a good night’s rest? | Female | 366 | 0.219 |  |
|  |  | Male | 378 |  |  |
|  |  | Total | 744 |  |  |
|  | Made you frustrated or annoyed about the amount of time you spend in the restroom? | Female | 366 | 0.347 |  |
|  |  | Male | 378 |  |  |
|  |  | Total | 744 |  |  |
|  | Made you avoid activities away from restrooms (i.e., walks, running, hiking)? | Female | 366 | 0.297 |  |
|  |  | Male | 378 |  |  |
|  |  | Total | 744 |  |  |
|  | Awakened you during sleep? | Female | 366 | 0.187 |  |
|  |  | Male | 378 |  |  |
|  |  | Total | 744 |  |  |
|  | Caused you to decrease your physical activities (exercising, sports, etc.)? | Female | 366 | 0.245 |  |
|  |  | Male | 378 |  |  |
|  |  | Total | 744 |  |  |
|  | Caused you to have problems with your partner or spouse? | Female | 366 | 0.161 |  |
|  |  | Male | 378 |  |  |
|  |  | Total | 744 |  |  |
|  | Made you uncomfortable while traveling with others because of needing to stop for a restroom? | Female | 366 | 0.893 |  |
|  |  | Male | 378 |  |  |
|  |  | Total | 744 |  |  |
|  | Affected your relationships with family and friends? | Female | 366 | **0.015** |  |
|  |  | Male | 378 |  |  |
|  |  | Total | 744 |  |  |
|  | Interfered with getting the amount of sleep you needed? | Female | 366 | **0.019** |  |
|  |  | Male | 378 |  |  |
|  |  | Total | 744 |  |  |
|  | Caused you embarrassment? | Female | 366 | 0.153 |  |
|  |  | Male | 378 |  |  |
|  |  | Total | 744 |  |  |
|  | Caused you to locate the closest restroom as soon as you arrive at a place you have never been? | Female | 366 | 0.081 |  |
|  |  | Male | 378 |  |  |
|  |  | Total | 744 |  |  |
| **Mann-Whitney U Test** | | | | | |

| Difference between current smoking in HRQL | | | | | | | | | | | | | | |
| --- | --- | --- | --- | --- | --- | --- | --- | --- | --- | --- | --- | --- | --- | --- |
| symptoms | | | None of the time | A little of the time | Some of the time | | A good bit of the time | Most of the time | | All of the time | | P value | |  |
| Caused you to plan ‘‘escape routes’’ to restrooms in public places? | No | 603 (63.2%) | | 186 (19.5%) | | 102 (10.7%) | 33 (3.5%) | | 23 (2.4%) | | 7 (0.7%) | | **0.045** |  |
|  | Yes | 23 (46.9%) | | 16 (32.7%) | | 6 (12.2%) | 2 (4.1%) | | 2 (4.1%) | |  | |  |  |
| Made you feel like there is something wrong with you? | No | 528 (55.3%) | | 215 (22.5%) | | 118 (12.4%) | 44 (4.6%) | | 28 (2.9%) | | 21 (2%) | | 0.798 |  |
|  | Yes | 25 (51%) | | 14 (28.6%) | | 7 (14.3%) | 1 (2%) | | 2 (4.1%) | |  | |  |  |
| Interfered with your ability to get a good night’s rest? | No | 538 (56.4%) | | 212 (22.2%) | | 114 (11.9%) | 40 (4.2%) | | 36 (3.8%) | | 14 (1.5%) | | 0.64 |  |
|  | Yes | 29 (59.2%) | | 10 (20.4%) | | 7 (14.3%) | 3 (6.1%) | |  | |  | |  |  |
| Made you frustrated or annoyed about the amount of time you spend in the restroom? | No | 561 (58.8%) | | 202 (21.1%) | | 110 (11.5%) | 46 (4.8%) | | 25 (2.6%) | | 10 (1%) | | 0.588 |  |
|  | Yes | 26 (53.1%) | | 14 (28.6%) | | 5 (10.2%) | 2 (4.1%) | | 1 (2%) | | 1 (2%) | |  |  |
| Made you avoid activities away from restrooms (i.e., walks, running, hiking)? | No | 688 (72.1%) | | 127 (13.3%) | | 77 (8.1%) | 35 (3.7%) | | 16 (1.7%) | | 11 (1.2%) | | 0.484 |  |
|  | Yes | 33 (67.3%) | | 8 (16.3%) | | 4 (4.1%) | 2 (4.1%) | | 2 (4.1%) | |  | |  |  |
| Awakened you during sleep? | No | 449 (47.1%) | | 285 (29.9%) | | 118 (12.4%) | 56 (5.9%) | | 35 (3.7%) | | 11 (1.2%) | | 0.367 |  |
|  | Yes | 20 (40.8%) | | 16 (14.3%) | | 7 (14.3%) | 1 (2%) | | 4 (8.2%) | | 1 (2%) | |  |  |
| Caused you to decrease your physical activities (exercising, sports, etc.)? | No | 710 (74.4%) | | 113 (11.8%) | | 77 (8.1%) | 24 (2.5%) | | 20 (2.1%) | | 10 (1%) | | 0.302 |  |
|  | Yes | 33 (67.3%) | | 8 (16.3%) | | 4 (8.2%) | 4 (8.2%) | |  | |  | |  |  |
| Caused you to have problems with your partner or spouse? | No | 793 (83.1%) | | 71 (7.4%) | | 50 (5.2%) | 23 (2.4%) | | 11 (1.2%) | | 6 (0.6%) | | 0.184 |  |
|  | Yes | 37 (75.5%) | | 6 (12.2%) | | 4 (8.2%) | 2 (4.1%) | |  | |  | |  |  |
| Made you uncomfortable while traveling with others because of needing to stop for a restroom? | No | 606 (63.5%) | | 176 (18.4%) | | 97 (10.2%) | 38 (4%) | | 22 (2.3%) | | 15 (1.6%) | | 0.621 |  |
|  | Yes | 30 (61.2%) | | 7 (14.3%) | | 8 (16.3%) | 3 (6.1%) | | 1 (2%) | |  | |  |  |
| Affected your relationships with family and friends? | No | 760 (79.7%) | | 88 (9.2%) | | 54 (5.7%) | 23 (2.4%) | | 21 (2.2%) | | 8 (0.8%) | | **0.038** |  |
|  | Yes | 33 (67.3%) | | 6 (12.2%) | | 6 (12.2%) | 3 (6.1%) | | 1 (2%) | |  | |  |  |
| Interfered with getting the amount of sleep you needed? | No | 623 (65.3%) | | 167 (17.5%) | | 83 (8.7%) | 45 (4.7%) | | 20 (2.1%) | | 16 (1.7%) | | 0.975 |  |
|  | Yes | 33 (67.3%) | | 5 (10.2%) | | 5 (10.2%) | 4 (8.2%) | | 1 (2%) | | 1 (2%) | |  |  |
| Caused you embarrassment? | No | 652 (68.3%) | | 149 (15.6%) | | 91 (9.5%) | 36 (3.8%) | | 15 (1.6%) | | 11 (1.2%) | | 0.934 |  |
|  | Yes | 33 (67.3%) | | 8 (16.3%) | | 6 (12.2%) | 1 (2%) | | 1 (2%) | |  | |  |  |
| Caused you to locate the closest restroom as soon as you arrive at a place you have never been? | No | 635 (66.6%) | | 159 (16.7%) | | 94 (9.9%) | 33 (3.5%) | | 21 (2.2%) | | 12 (1.3%) | | 0.81 |  |
|  | Yes | 34 (69.4%) | | 5 (10.2%) | |  | 8 (16.3%) | | 1 (2%) | | 1 (2%) | |  |  |
| Mann-Whitney U Test | | | | | | | | | | | | | | |

| Difference between Energy drink status in HRQL | | | | | | | | | |
| --- | --- | --- | --- | --- | --- | --- | --- | --- | --- |
| symptoms | | None of the time | A little of the time | Some of the time | A good bit of the time | Most of the time | All of the time | P value |  |
| Caused you to plan ‘‘escape routes’’ to restrooms in public places? | No | 532 (63.6%) | 161 (19.2%) | 90 (10.8%) | 28 (3.3%) | 19 (2.3%) | 7 (0.8%) | 0.133 |  |
|  | Yes | 94 (56.6%) | 41 (24.7%) | 18 (10.8%) | 7 (4.2%) | 6 (3.6%) |  |  |  |
| Made you feel like there is something wrong with you? | No | 463 (55.3%) | 188 (22.5%) | 105 (12.5%) | 43 (5.1%) | 22 (2.6%) | 16 (1.9%) | 0.884 |  |
|  | Yes | 90 (54.2%) | 41 (24.7%) | 20 (12%) | 2 (1.2%) | 8 (4.8%) | 5 (3%) |  |  |
| Interfered with your ability to get a good night’s rest? | No | 492 (58.8%) | 177 (21.1%) | 96 (11.5%) | 34 (4.1%) | 27 (3.2%) | 11 (1.3%) | **0.001** |  |
|  | Yes | 75 (45.2%) | 45 (27.1%) | 25 (15.1%) | 9 (5.4%) | 9 (5.4%) | 3 (1.8%) |  |  |
| Made you frustrated or annoyed about the amount of time you spend in the restroom? | No | 496 (59.3%) | 176 (21%) | 95 (11.4.%) | 42 (5%) | 20 (2.4%) | 8 (1%) | 0.324 |  |
|  | Yes | 91 (54.8%) | 40 (24.1%) | 20 (12%) | 6 (3.6%) | 6 (3.6%) | 3 (1.8%) |  |  |
| Made you avoid activities away from restrooms (i.e., walks, running, hiking)? | No | 621 (74.2%) | 104 (12.4%) | 62 (7.4%) | 31 (3.7%) | 12 (1.4%) | 7 (0.8%) | **<0.001** |  |
|  | Yes | 100 (60.2%) | 31 (18.7%) | 19 (11.4%) | 6 (3.6%) | 6 (3.6%) | 4 (2.4%) |  |  |
| Awakened you during sleep? | No | 409 (48.9%) | 247 (29.5%) | 93 (11.1%) | 49 (5.9%) | 29 (3.5%) | 10 (1.2%) | **0.002** |  |
|  | Yes | 60 (36.1%) | 54 (32.5%) | 32 (19.3%) | 8 (4.8%) | 10 (6%) | 2 (1.2%) |  |  |
| Caused you to decrease your physical activities (exercising, sports, etc.)? | No | 634 (75.7%) | 101 (12.1%) | 60 (7.2%) | 20 (2.4%) | 14 (1.7%) | 8 (1%) | **0.003** |  |
|  | Yes | 109 (65.7%) | 20 (12%) | 21 (12.7%) | 8 (4.8%) | 6 (3.6%) | 2 (1.2%) |  |  |
| Caused you to have problems with your partner or spouse? | No | 699 (83.5%) | 64 (7.6%) | 44 (5.3%) | 15 (1.8%) | 8 (1%) | 7 (0.8%) | 0.119 |  |
|  | Yes | 131 (78.9%) | 13 (7.8%) | 10 (6%) | 8 (4.8%) | 3 (1.8%) | 1 (0.6%) |  |  |
| Made you uncomfortable while traveling with others because of needing to stop for a restroom? | No | 542 (64.8%) | 154 (9.6%) | 80 (9.6%) | 31 (3.7%) | 20 (2.4%) | 10 (1.2%) | **0.019** |  |
|  | Yes | 94 (56.6%) | 29 (15.1%) | 25 (15.1%) | 10 (6%) | 3 (1.8%) | 5 (3%) |  |  |
| Affected your relationships with family and friends? | No | 677 (80.9%) | 74 (8.8%) | 43 (5.1%) | 17 (2%) | 18 (2.2%) | 8 (1%) | **0.001** |  |
|  | Yes | 116 (69.9%) | 20 (12%) | 17 (10.2%) | 9 (5.4%) | 4 (2.4%) |  |  |  |
| Interfered with getting the amount of sleep you needed? | No | 560 (66.9%) | 142 (17%) | 71 (8.5%) | 36 (4.3%) | 13 (1.6%) | 15 (1.8%) | **0.011** |  |
|  | Yes | 96 (57.8%) | 30 (18.1%) | 17 (10.2%) | 13 (7.8) | 8 (4.8%) | 2 (1.2%) |  |  |
| Caused you embarrassment? | No | 585 (69.9%) | 123 (14.7%) | 78 (9.3%) | 30 (3.6%) | 11 (1.3%) | 10 (1.2%) | **0.020** |  |
|  | Yes | 100 (60.2%) | 34 (20.5%) | 19 (11.4%) | 7 (4.2%) | 4 (2.4%) | 2 (1.2%) |  |  |
| Caused you to locate the closest restroom as soon as you arrive at a place you have never been? | No | 569 (68%) | 130 (15.5%) | 84 (10%) | 26 (3.1%) | 18 (2.2%) | 10 (1.2%) | 0.067 |  |
|  | Yes | 100 (60.2%) | 34 (20.5%) | 18 (10.8%) | 7 (4.2%) | 4 (2.4%). | 3 (1.8%) |  |  |
| Mann-Whitney U Test | | | | | | | | |  |

| Difference between Coffee drink status in HRQL | | | | | | | | | |
| --- | --- | --- | --- | --- | --- | --- | --- | --- | --- |
| symptoms | | None of the time | A little of the time | Some of the time | A good bit of the time | Most of the time | All of the time | P value |  |
| Caused you to plan ‘‘escape routes’’ to restrooms in public places? | No | 299 (66%) | 74 (16.3%) | 49 (10.8%) | 16 (3.5%) | 12 (2.6%) | 3 (0.7%) | 0.099 |  |
|  | Yes | 327 (59.5%) | 128 (23.3%) | 59 (10.7%) | 19 (3.5%) | 13 (2.4%) | 4 (0.7%) |  |  |
| Made you feel like there is something wrong with you? | No | 260 (57.4%) | 104 (23%) | 43 (9.5%) | 27 (6%) | 12 (2.6%) | 7 (1.5%) | 0.153 |  |
|  | Yes | 293 (53.3%) | 125 (22.7%) | 82 (14.9%) | 18 (3.3%) | 18 (3.3%) | 14 (2.5%) |  |  |
| Interfered with your ability to get a good night’s rest? | No | 260 (57.4%) | 99 (21.9%) | 52 (11.5%) | 17 (3.8%) | 18 (4%) | 7 (1.5%) | 0.645 |  |
|  | Yes | 307 (55.8%) | 123 (22.4%) | 69 (12.5%) | 26 (4.7%) | 18 (3.3%) | 7 (1.3%) |  |  |
| Made you frustrated or annoyed about the amount of time you spend in the restroom? | No | 275 (60.7%) | 87 (19.2%) | 48 (10.6%) | 26 (5.7%) | 12 (2.6%) | 5 (1.1%) | 0.392 |  |
|  | Yes | 312 (56.7%) | 129 (23.5%) | 67 (12.2%) | 22 (4%) | 14 (2.5%) | 6 (1.1%) |  |  |
| Made you avoid activities away from restrooms (i.e., walks, running, hiking)? | No | 331 (73.1%) | 64 (14.1%) | 30 (6.6%) | 13 (2.9%) | 10 (2.2%) | 5 (1.1%) | 0.367 |  |
|  | Yes | 390 (70.9%) | 71 (12.9%) | 51 (9.3) | 24 (4.4%) | 8 (1.5%) | 6 (1.1%) |  |  |
| Awakened you during sleep? | No | 238 (52.5%) | 120 (26.5%) | 50 (11%) | 25 (5.5%) | 14 (3.1%) | 6 (1.3%) | **0.003** |  |
|  | Yes | 231 (42%) | 181 (32.9%) | 75 (13.6%) | 32 (5.8%) | 25 (4.5%) | 6 (1.1%) |  |  |
| Caused you to decrease your physical activities (exercising, sports, etc.)? | No | 343 (75.7%) | 59 (13%) | 30 (6.6%) | 10 (2.2%) | 7 (1.5%) | 4 (0.9%) | 0.176 |  |
|  | Yes | 400 (72.7%) | 62 (11.3%) | 51 (9.3%) | 18 (3.3%) | 13 (2.4%) | 6 (1.1%) |  |  |
| Caused you to have problems with your partner or spouse? | No | 380 (83.9%) | 37 (8.2%) | 24 (5.3%) | 6 (1.3%) | 5 (1.1%) | 1 (0.2%) | 0.295 |  |
|  | Yes | 450 (81.8%) | 40 (7.3%) | 30 (5.5%) | 17 (3.1%) | 6 (1.1%) | 7 (1.3%) |  |  |
| Made you uncomfortable while traveling with others because of needing to stop for a restroom? | No | 296 (65.3%) | 86 (19%) | 39 (8.6%) | 20 (4.4%) | 8 (4.4%) | 4 (0.9%) | 0.138 |  |
|  | Yes | 340 (61.8%) | 97 (12%) | 66 (12%) | 21(3.8%) | 15 (3.8%) | 11 (2%) |  |  |
| Affected your relationships with family and friends? | No | 368 (81.2%) | 35 (7.7%) | 22 (4.9%) | 12 (2.6%) | 13 (2.6%) | 3 (0.7%) | 0.176 |  |
|  | Yes | 425 (77.3%) | 59 (10.7%) | 38 (6.9%) | 14 (5.5%) | 9 (2.5%) | 5 (0.9%) |  |  |
| Interfered with getting the amount of sleep you needed? | No | 301 (66.4%) | 79 (17.4%) | 34 (7.5%) | 24 (5.3%) | 7 (5.3%) | 8 (1.9%) | 0.463 |  |
|  | Yes | 355 (64.5%) | 93 (16.9%) | 54 (9.8%) | 25 (4.5%) | 14 (4.5%) | 9 (1.6%) |  |  |
| Caused you embarrassment? | No | 321 (70.9%) | 64 (14.1%) | 40 (8.8%) | 16 (3.5%) | 5 (3.5%) | 7 (1.5%) | 0.140 |  |
|  | Yes | 364 (66.2%) | 93 (16.9%) | 57 (10.4%) | 21 (3.8%) | 10 (3.8%) | 5 (0.9%) |  |  |
| Caused you to locate the closest restroom as soon as you arrive at a place you have never been? | No | 316 (69.8%) | 64 (14.1%) | 41 (9.1%) | 14 (3.1%) | 12 (3.1%) | 6 (1.3%) | 0.108 |  |
|  | Yes | 353 (64.2%) | 100 (18.2%) | 61 (11.1%) | 19 (3.5%) | 10 (3.5%) | 7 (1.3%) |  |  |
| Mann-Whitney U Test | | | | | | | | |  |

| Difference between Tea drink status in HRQL | | | | | | | | | |
| --- | --- | --- | --- | --- | --- | --- | --- | --- | --- |
| symptoms | | None of the time | A little of the time | Some of the time | A good bit of the time | Most of the time | All of the time | P value |  |
| Caused you to plan ‘‘escape routes’’ to restrooms in public places? | No | 216 (66.3%) | 56 (17.2%) | 28 (8.6%) | 16 (4.9%) | 8 (2.5%) | 2 (0.6%) | 0.150 |  |
|  | Yes | 410 (60.6%) | 146 (21.6%) | 80 (11.8%) | 19 (2.8%) | 17 (2.5%) | 5 (0.7%) |  |  |
| Made you feel like there is something wrong with you? | No | 189 (58%) | 65 (19.9%) | 36 (11%) | 18 (5.5%) | 10 (3.1%) | 8 (2.5%) | 0.425 |  |
|  | Yes | 364 (53.8%) | 164 (24.2%) | 89 (13.1%) | 27 (4%) | 20 (3%) | 13 (1.9%) |  |  |
| Interfered with your ability to get a good night’s rest? | No | 186 (57.1%) | 71 (21.8%) | 41 (12.6%) | 11 (3.4%) | 10 (3.1%) | 7 (2.1%) | 0.823 |  |
|  | Yes | 381 (56.3%) | 151 (22.3%) | 80 (11.8%) | 32 (4.7%) | 26 (3.8%) | 7 (1%) |  |  |
| Made you frustrated or annoyed about the amount of time you spend in the restroom? | No | 195 (59.8%) | 67 (20.6%) | 38 (11.7%) | 14 (4.3%) | 7 (2.1%) | 5 (1.5%) | 0.607 |  |
|  | Yes | 392 (57.9%) | 149 (22%) | 77 (11.4%) | 34 (5%) | 19 (2.8%) | 6 (0.9%) |  |  |
| Made you avoid activities away from restrooms (i.e., walks, running, hiking)? | No | 239 (73.3%) | 48 (14.7%) | 19 (5.8%) | 10 (3.1%) | 5 (1.5%) | 5 (1.5%) | 0.379 |  |
|  | Yes | 482 (71.2%) | 87 (12.9%) | 62 (9.2%) | 27 (4%) | 13 (1.9%) | 6 (0.9%) |  |  |
| Awakened you during sleep? | No | 165 (50.6%) | 86 (26.4%) | 40 (12.3%) | 22 (6.7%) | 8 (2.5%) | 5 (1..5%) | 0.199 |  |
|  | Yes | 304 (44.9%) | 215 (31.8%) | 85 (12.6%) | 35 (5.2%) | 31 (4.6%) | 7 (1%) |  |  |
| Caused you to decrease your physical activities (exercising, sports, etc.)? | No | 248 (76.1%) | 38 (11.7%) | 25 (7.7%) | 8 (2.5%) | 6 (1.8%) | 1 (0.3%) | 0.270 |  |
|  | Yes | 495 (73.1%) | 83 (12.3%) | 56 (8.3%) | 20 (3%) | 14 (2.1%) | 9 (1.3%) |  |  |
| Caused you to have problems with your partner or spouse? | No | 273 (83.7%) | 24 (7.4%) | 14 (4.3%) | 8 (2.5%) | 5 (1.5%) | 2 (0.6%) | 0.584 |  |
|  | Yes | 557 (82.3%) | 53 (7.8%) | 40 (5.9%) | 15 (2.2%) | 6 (0.9%) | 6 (0.9%) |  |  |
| Made you uncomfortable while traveling with others because of needing to stop for a restroom? | No | 217 (66.6%) | 57 (17.5%) | 26 (8%) | 15 (4.6%) | 7 (2.1%) | 4 (1.2%) | 0.146 |  |
|  | Yes | 419 (61.9%) | 126 (18.6%) | 79 (11.7%) | 26 (3.8%) | 16 (2.4%) | 11 (1.6%) |  |  |
| Affected your relationships with family and friends? | No | 262 (80.4%) | 28 (8.6%) | 16 (4.9%) | 8 (2.5%) | 9 (2.8%) | 3 (0.9%) | 0.538 |  |
|  | Yes | 531 (78.4%) | 66 (9.7%) | 44 (6.5%) | 18 (2.7%) | 13 (1.9%) | 5 (0.7%) |  |  |
| Interfered with getting the amount of sleep you needed? | No | 226 (69.3%) | 48 (14.7%) | 23 (7.1%) | 16 (4.9%) | 6 (1.8%) | 7 (2.1%) | 0.109 |  |
|  | Yes | 430 (63.5%) | 124 (18.3%) | 65 (9.6%) | 33 (4.9%) | 15 (2.2%) | 10 (1.5%) |  |  |
| Caused you embarrassment? | No | 227 (69.6%) | 44 (13.5%) | 33 (10.1%) | 12 (3.7%) | 4 (1.2%) | 6 (1.8%) | 0.701 |  |
|  | Yes | 458 (67.7%) | 113 (16.7%) | 64 (9.5%) | 25 (3.7%) | 11 (1.6%) | 6 (0.9%) |  |  |
| Caused you to locate the closest restroom as soon as you arrive at a place you have never been? | No | 224 (68.7%) | 53 (16.3%) | 28 (8.6%) | 9 (2.8%) | 9 (2.8%) | 3 (0.9%) | 0.310 |  |
|  | Yes | 445 (65.7%) | 111 (16.4%) | 74 (10.9%) | 24 (3.5%) | 13 (1.9%) | 10 (1.5%) |  |  |
| Mann-Whitney U Test | | | | | | | | |  |

**Correlation**

| **Confidence Intervals of Spearman's rho** | | | | | |
| --- | --- | --- | --- | --- | --- |
|  | Spearman's rho | P value | 95% CI | | Correlation coefficient |
|  |  |  | Lower | Upper |  |
| OAB–HRQL | -0.708 | <0.001 | -0.738 | -0.674 | -0.708 |

| **Linear Regression on the effect of OAB bother on the HRQL** | | | | | | | | | |
| --- | --- | --- | --- | --- | --- | --- | --- | --- | --- |
| Model | Unstandardized Coefficients | | Standardized Coefficients | t | P value | 95% CI for B | Regression P value | R | R^2^ |
|  | B | Std. Error | Beta |  |  |  |  |  |  |
| (Constant) | 98.296 | 0.518 |  | 189.74 | <0.001 | (97.280, 99.313) | <0.001 | 0.692 | 0.479 |
| OAB bother | -0.660 | 0.022 | -0.692 | -30.338 | **<0.001** | (-0.702, -0.617) |  |  |  |

| **Multiple linear regression assessing the effect of several risk factors on overactive bladder symptoms, bother, and their related quality of life** | | | | | | |
| --- | --- | --- | --- | --- | --- | --- |
| **Overactive bladder symptoms bother (OAB)** | | | | | | |
| Model | Unstandardized Coefficients | | Standardized Coefficients | t | P value | 95% CI for B |
|  | B | Std. Error | β |  |  |  |
| (Constant) | 2.066 | 8.558 |  | 0.241 | 0.809 | (-14.73, 18.7) |
| Age | 0.541 | 0.437 | 0.054 | 1.238 | 0.216 | (-0.317, 1.4) |
| Gender (ref: female) | 0.836 | 1.105 | 0.025 | 0.757 | 0.449 | (-1.332, 3) |
| Academic phase (ref: academic) | -5.448 | 1.670 | -0.141 | -3.262 | **0.001*** | (-8.726, -2.171) |
| BMI | 0.659 | 0.741 | 0.028 | 0.889 | 0.374 | (-0.795, 2.112) |
| Rate your satisfaction with your academic performance | 0.466 | 0.641 | 0.025 | 0.726 | 0.468 | (-0.793, 1.725) |
| Rate your satisfaction with your social life | 1.327 | 0.650 | 0.072 | 2.040 | **0.042*** | (0.051, 2.603) |
| Rate your stress level | 1.259 | 0.497 | 0.083 | 2.535 | **0.011*** | (0.284, 2.234) |
| smoking | -2.331 | 2.576 | -0.030 | -0.905 | 0.366 | (-7.387, 2.725) |
| Energy drinks (ref: No) | 3.045 | 1.528 | 0.067 | 1.992 | **0.047*** | (0.046, 6.043) |
| Coffee (ref: No) | 0.727 | 1.131 | 0.021 | 0.643 | 0.520 | (-1.492, 2.946) |
| Tea (ref: No) | -0.668 | 1.168 | -0.018 | -0.572 | 0.568 | (-2.960, 1.625) |
| **Health-related quality of life (HRQL)** | | | | | | |
| (Constant) | 112.850 | 5.961 |  | 18.932 | <0.001 | (101.15, 124.55) |
| OAB bother | -0.655 | 0.022 | -0.687 | -29.582 | **<0.001*** | (-0.698, -0.611) |
| Age | -0.714 | 0.305 | -0.075 | -2.340 | **0.019*** | (-1.312, -0.115) |
| Gender (ref: female) | -0.689 | 0.770 | -0.021 | -0.895 | 0.371 | (-2.199, 0.822) |
| Academic phase (ref: Academic) | 2.745 | 1.170 | 0.075 | 2.347 | **0.019*** | (0.45, 5.04) |
| BMI | -0.013 | 0.516 | -0.001 | -0.026 | 0.979 | (-1.026, 0.999) |
| Rate your satisfaction with your academic performance | 0.144 | 0.447 | 0.008 | 0.321 | 0.748 | (-0.733, 1.021) |
| Rate your satisfaction with your social life | 0.339 | 0.454 | 0.019 | 0.746 | 0.456 | (-0.552, 1.230) |
| Rate your stress level | -0.155 | 0.347 | -0.011 | -0.447 | 0.655 | (-0.836, 0.526) |
| Smoking (ref: No) | -0.552 | 1.795 | -0.007 | -0.307 | 0.759 | (-4.075, 2.971) |
| Energy drinks (ref: No) | -1.072 | 1.067 | -0.025 | -1.005 | 0.315 | (-3.165, 1.021) |
| Coffee (ref: No) | -0.188 | 0.788 | -0.006 | -0.239 | 0.811 | (-1.734, 1.357) |
| Tea (ref: No) | -0.911 | 0.814 | -0.026 | -1.119 | 0.263 | (-2.507, 0.686) |

| **Model fit** | | | | | |
| --- | --- | --- | --- | --- | --- |
| Model | Regression P value | R | R^2^ | Adjusted R^2^ | SE of the Estimate |
| OAB | **<0.001** | 0.193 | 0.037 | 0.026 | 16.69 |
| HRQL | **<0.001** | 0.697 | 0.486 | 0.480 | 11.63 |

**Dependent variables**: OAB and HRQL in each model

**Independent variables:** Age, sex, BMI, academic phase, satisfaction with academic performance, and social life. Stress level, smoking, energy drinks, and both coffee and tea

Coding for each categorical data: The reference group was coded 1, while the other group was coded 2. Ex: gender : reference group was female (code: 1), and the other group was the male group (code 2)
